# Identification of stage-associated exosome miRNAs in colorectal cancer by improved robust and corroborative approach embedded miRNA-target network

**DOI:** 10.3389/fmed.2022.881788

**Published:** 2022-09-27

**Authors:** Fei Long, Luyao Tian, Zixuan Chai, Jing Li, Ying Tang, Mingwei Liu

**Affiliations:** Key Laboratory of Clinical Laboratory Diagnostics, College of Laboratory Medicine, Chongqing Medical University, Chongqing, China

**Keywords:** colorectal cancer, exosome, miRNA, stage, network analysis

## Abstract

**Background:**

Colorectal cancer (CRC) is a common gastrointestinal tumor with high morbidity and mortality. At the molecular level, patients at different stages present considerable heterogeneity. Although the miRNA in exosome is an effective biomarker to reveal tumor progression, studies based on stage-associated exosome miRNA regulatory network analysis still lacking. This study aims to identify CRC stage-associated exosome miRNAs and reveal their potential function in tumor progression.

**Methods:**

In this study, serum and cellular exosome miRNA expression microarrays associated with CRC were downloaded from GEO database. Stage-common (SC) and stage-specific (SS) differentially expressed miRNAs were extracted and their targets were identified based on 11 databases. Furthermore, miRNA SC and SS regulatory function networks were built based on the CRC phenotypic relevance of miRNA targets, and the corresponding transcription factors were identified. Concurrently, the potential stage-associated miRNAs were identified by receiver-operating characteristic (ROC) curve analysis, survival analysis, drug response analysis, ceRNA analysis, pathway analysis and a comprehensive investigation of 159 publications.

**Results:**

Ten candidate stage-associated miRNAs were identified, with three SC (miR-146a-5p, miR-22-3p, miR-23b-3p) and seven SS (I: miR-301a-3p, miR-548i; IIIA: miR-23a-3p; IV: miR-194-3p, miR-33a-3p, miR-485-3p, miR-194-5p) miRNAs. Additionally, their targets were enriched in several vital cancer-associated pathways such as TGF-beta, p53, and hippo signaling pathways. Moreover, five key hotspot target genes (*CCNA2, MAPK1, PTPRD, MET*, and *CDKN1A*) were demonstrated to associated with better overall survival in CRC patients. Finally, miR-23b-3p, miR-301a-3p and miR-194-3p were validated being the most stably expressed stage-associated miRNAs in CRC serum exosomes, cell exosomes and tissues.

**Conclusions:**

These CRC stage-associated exosome miRNAs aid to further mechanism research of tumor progression and provide support for better clinical management in patients with different stages.

## Introduction

Colorectal cancer (CRC) is an aggressive and fatal malignancy that develops from normal intestinal cells, with up to 1.85 million new cases diagnosed each year worldwide. The mortality rate is as high as 45.9% (850,000 cases) (1), which makes it the world's third most common and second-deadliest cancer (2). CRC is characterized by insidious and rapid progression, metastasis, and treatment difficulty in the middle and late stages. Previous studies have shown that high inter-patient and intra-tumor heterogeneity promotes CRC metastasis and patient deterioration (3). Exosomes have been discovered to be a powerful screening and diagnostic tool for highly heterogeneous malignancies, including CRC, due to their widespread presence in blood, urine, cerebrospinal fluid, and other body fluids, with the outstanding advantages of richer molecules, better representation, higher stability, and greater clinical applicability (4–8). Exosome miRNAs (exo-miRNAs), the cancer genome dark matter, maybe suitable biomarkers for cancer screening, stage monitoring and precise treatment (9, 10). Furthermore, exosome miRNAs have a dramatic impact on metastasis, progression, prognosis and regulation of biological networks in CRC patients.

For instance, Matsumura et al. (11) discovered that serum exosome miR-17-92a cluster expression level was correlated with CRC progression and miR-19a with the prognostic significance of expression might be a prognostic biomarker for recurrence in CRC patients. The miR-21 was reported to be highly expressed in various CRC cell exosomes, plasma exosomes and tissues, and correlated with disease staging, liver metastasis and prognosis, which could be used as a reference marker for gastrointestinal cancers (12). Nearly a hundred exosome miRNAs, such as miR-23a, miR-92a, miR-1246, and miR-486, have been identified as promising biomarkers for CRC (13–17). However, the correlation of exosome miRNAs with CRC staging and how it affects CRC progression is less understood. Identification of stage-associated exosome miRNA biomarkers is critical to facilitate further mechanistic studies and dissect tumor progression.

It is encouraging that untargeted omics data and text-derived knowledge have improved biomarker identification. The omics-driven and network-based approaches, composed of biological and computational processes, can lead to the more precise discovery of potential novel biomarkers due to corroborative evidence integrated with interacting molecular entities and incorporating measurements of molecule expression and biological function (18–21). The miRNA targeting topology networks are frequently used to extract key miRNAs from noisy backgrounds for further translational studies (22, 23). In contrast to this approach, Chen et al. (24) increased our understanding of miRNA biology by developing a single-line regulatory miRNA vulnerability network and identifying the corresponding biomarkers. However, single network analysis is insufficient, and the integration of diverse data (e.g., disease relevance of target molecules, ability to regulate transcription factors, and biological pathways) can aid in identifying the stage-associated biomarkers.

**Figure 1:**
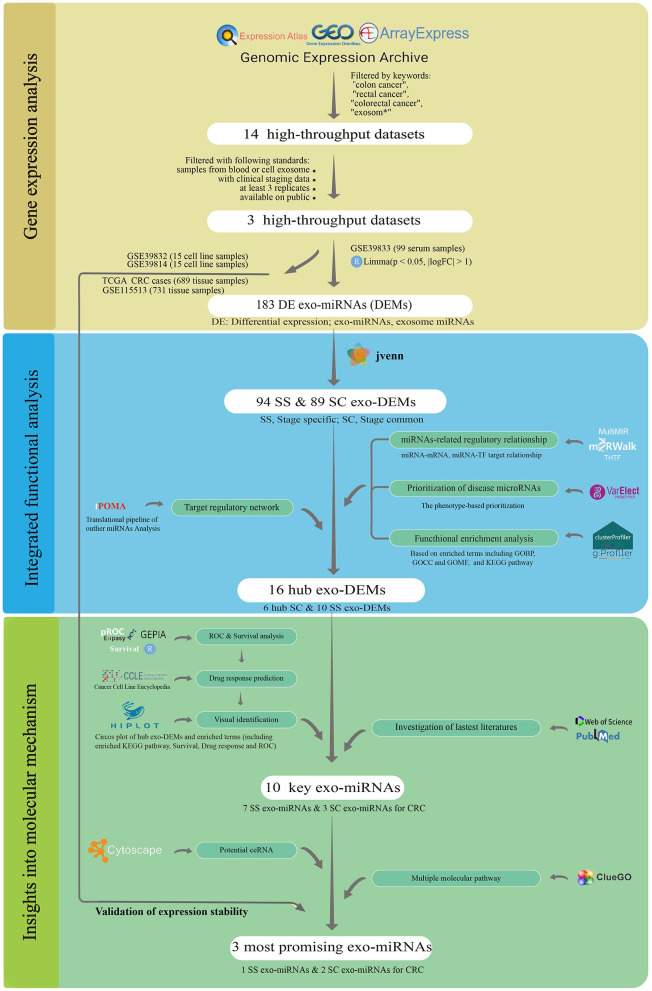
The workflow of this study.

Here, we systematically presented an enhanced analysis with a robust and corroborative workflow to identify stage-associated exo-miRNAs biomarkers. In this study, the miRNA single-line regulation vulnerability network construction pipeline was proposed and combined with the marker robust analysis strategy proposed in our previous work (25). As illustrated in Figure 1, three CRC serum and cell exosome datasets were first collected from four public databases. Subsequently, stage-common (SC) and stage-specific (SS) miRNA targeting topology networks for CRC were built, and their distinct biological functions were elucidated. Ten key stage-associated miRNA were further selected from different networks and assessed by receiver operating characteristic (ROC) curve analysis. Additionally, survival analysis and drug response analysis were performed for them at the tissue and cellular levels. Moreover, the competing endogenous RNA (ceRNA) networks were constructed, and the targeting pathways were explored. Finally, three miRNAs were validated presenting stably expression in serum exosomes, cellular exosomes, and tumor tissues. The multi-level and multi-information integration approach enables a robust analysis for biomarker discovery.

## Materials and methods

### Collection and filtering of exosome data sets

To obtain complete datasets of exosome miRNA (exo-miRNA) related gene expression in CRC, four public databases were retrieved using the keywords “colon cancer,” “rectal cancer,” “colorectal cancer,” “exosome,” “exosomes,” and “exosomal.” The databases Gene Expression Omnibus (GEO, http://www.ncbi.nlm.nih.gov/geo) (26), ArrayExpress (https://www.ebi.ac.uk/arrayexpress), ExpressionAtlas (https://www.ebi.ac.uk/gxa), and Genomic Expression Archive (GEA, https://www.ddbj.nig.ac.jp/gea) (Supplementary Table 1). Afterward, the following criteria were used to filter the data: (i) the data objects were miRNA expression profiles in blood or cellular exosomes. (ii) The blood exosome samples contained clinical staging information. (iii) At least three sample replicates. (iv) They were completely free and downloadable. Finally, three CRC exosome datasets were obtained: one associated with serum exosomes (GSE39833), which included 20 patients with stage I, 20 patients with stage II, 20 patients with stage IIIA, 16 patients with stage IIIB, 12 patients with stage IV, and 11 healthy individuals; the other two were associated with CRC cell line exosomes (GSE39832 and GSE39814), which included four fetal bovine serum-treated controls, three normal colon cells (FHC) and 15 CRC cells (HCT116, HT29, RKO, SW48, SW480; three of each).

### Transcriptome data pre-processing and differentially expressed exo-miRNA identification

Background-corrected and normalized Agilent microarray expression matrixes from the datasets above were downloaded, negative values were removed, and interpolation was performed using the caret R package's preProcess function (27). All values were log2-transformed. The limma R package (28) was used to analyze differentially expressed miRNAs in serum exosomes from different stages of CRC. Differential significance was determined with *p* < 0.05 and |logFC| > 1 (Supplementary Figure 1), and differential intersection screening and definition were performed by Jvenn online tool (29). MiRBase 22.1 (http://www.mirbase.org/index.shtml) was used to transform the names of all miRNAs obtained. Heatmap data were normalized using the scale function, clustered using fully linked hierarchical clustering based on Euclidean distances (if necessary), and visualized by the pheatmap R package (30).

To determine exo-miRNA target gene expression and miRNA expression in tissues, the GDC-TCGA (31) colon adenocarcinoma (COAD) and rectal adenocarcinoma (READ) log2(count+1)-transformed mRNA expression matrix, log2(RPM+1)-transformed miRNA expression matrix and the corresponding clinical data were downloaded from UCSC-ZENA (https://xenabrowser.net/datapages/) (32). COAD included 41 normal samples and 471 samples from colon cancer patients and READ included 10 normal samples and 167 samples from rectal cancer patients. Moreover, dataset GSE115513 (33) was used to validate the miRNA expression in the tissue level, which included 649 normal intestinal mucosa samples and 731 CRC tumor tissue samples (including 231 in stage I, 181 in stage II, 219 in stage III, and 100 in stage IV). The normalized expression matrix was downloaded from GEO and log2 transformed. Key miRNAs were selected and compared among different stages, the Wilcoxon test was used to evaluate the differences between two stages and the Kruskal-Wallis test was applied to estimate the differences among all stages. Gene expression and drug sensitivity data of 22 CRC cell lines were downloaded from the CCLE database (34).

### Prediction and screening of targeted genes from differentially expressed exo-miRNAs

To obtain the targeted genes from differentially expressed exo-miRNAs, 11 databases were searched based on the multiMiR R package (35) and miRWalk 2.0 (36), including three experimental validation databases [miRecords 4.0 (37), mirTarBase 7.0 (38) and Tarbase 8.0 (39)] and eight prediction databases [DIANA-microT-CDS 5.0 (40), EIMMo 5.0 (41), MicroCosm 5.0 (http://www.ebi.ac.uk/enright-srv/microcosm/cgi-bin/targets/v5/download.pl), miRanda (42), miRDB 6.0 (43), PicTar 2.0 (44), PITA 6.0 (45) and TargetScan 7.2 (46)]. The top 20% of data with high confidence based on *p*-value and included in at least 4 (5,127 miRNA-gene pairs) of the 8 (133,738 miRNA-gene pairs) prediction databases were taken. miRanda, PITA 6.0, and TargetScan 7.2 were specifically chosen to include only predictions with conserved loci. Furthermore, data from at least 2 (2,825 miRNA-gene pairs) of the 3 (46,455 miRNA-gene pairs) experimental validation databases were chosen. Finally, 7,306 miRNA-gene pairs were obtained from the 11 databases. The accuracy of prediction and the richness of miRNA targeting prediction results were ensured by combining experimental validation databases and prediction databases.

The VarElect online tool was used to perform phenotypic correlation analysis based on the keywords “Colon Cancer” OR “Rectal Cancer” OR “Colorectal Cancer” to further screen for target genes associated with CRC (47). Phenotype scores greater than five, and only direct phenotypic associations were considered.

### Exosomal miRNA targeting analysis process establishment

**Figure 2:**
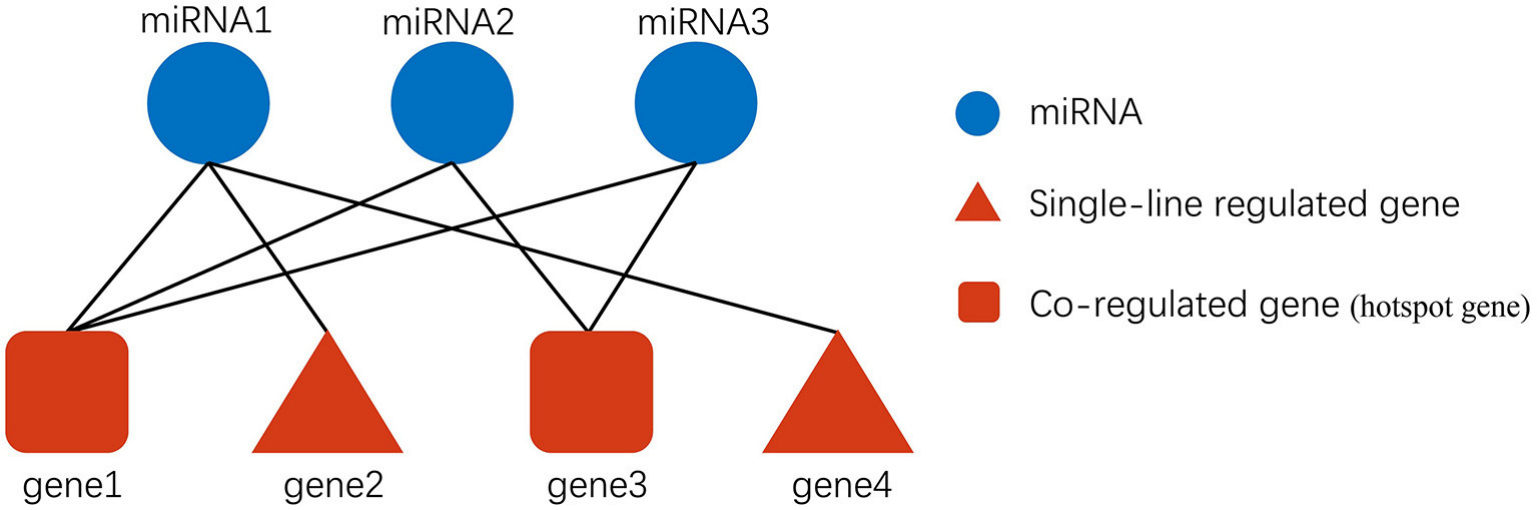
Description of exosomal miRNA-gene regulation. Gene2 and gene4 are only regulated by miRNA1. They are single-line regulated by miRNA, and if a specific miRNA single-line regulates more genes, then the miRNA may be more important. In addition, gene1 is regulated by miRNA1, miRNA2, and miRNA3 simultaneously, so gene1 may be more susceptible to be influenced by exosomal miRNAs than gene3.

The Pipeline of Outlier miRNA Analysis (POMA) is an analytical process developed by Chen et al. (24) for identifying key miRNAs. A translational POMA (tPOMA) in exo-miRNA was created to identify key exo-miRNAs in CRC (Figure 2). tPOMA is constructed based on two key assumptions: (i) The number of targeted CRC-related genes/TFs can reflect the regulatory activity of CRC exo-miRNAs. (ii) If only one exo-miRNA target a CRC-related gene/TF, that exo-miRNA is more likely to show regulatory specificity to that gene/TF. Thus, the greater the number of CRC-related genes/TFs regulated by an exo-miRNA in a single line, the more important that miRNA is likely to be. Furthermore, we suppose many CRC exo-miRNAs regulate a CRC-related gene. In that case, the gene is more likely to be repressed (miRNAs typically repress gene expression), and such a gene was referred to as a hotspot gene regulated by exo-miRNAs.

Based on this assumption, a tPOMA-based screening was performed in four steps on all obtained SC exo-miRNAs: (i) First, a global targeting network of SC exo-miRNAs was built using the previously mentioned 11 databases. (ii) The miRNA single-line regulatory network was further constructed, in which genes only regulated by one exo-miRNA (other exo-miRNAs do not regulate the gene). (iii) To generate a hotspot gene network, single-line regulated genes were removed from the global miRNA-gene network (genes are regulated by at least two exo-miRNAs). (iv) Using the VarElect tool, CRC phenotype-related genes were selected to built the CRC-related single-line regulated gene network and hotspot gene network (47). (v) The TF was confirmed using the THTF database (http://humantfs.ccbr.utoronto.ca) (48). Finally, the network of exo-miRNAs targeting genes was mapped using Cytoscape (3.7.1) (49).

The online tool miRNet (50) developed by Chang et al. was used to construct a target network of SC exo-miRNAs (target molecules including mRNAs, lncRNAs, circRNAs, pseudogenes, and sncRNAs) and ranked the miRNAs by the degree to demonstrate the reliability of our tPOMA model. The top 10 miRNAs were compared to the top 10 miRNAs predicted by our tPOMA model (Supplementary Figure 2), and there is a high degree of overlap (6 out of 10 miRNAs were consistent).

The following three steps were used to build the SS exo-miRNA subnetworks: (i) The global network of SS exo-miRNAs and target genes was built using the results of the previously mentioned 11 databases. (ii) Genes only present at a specific stage were extracted by Jvenn. (iii) SS exo-miRNAs and target gene sub-networks were further constructed by Cytoscape.

### Enrichment analysis of biological functions and pathways

To better understand the role of these targeted genes in the development of CRC, enrichment analysis of Gene Ontology (GO) (51) and Kyoto Encyclopedia of Genes and Genomes (KEGG) (52) was performed by the clusterProfiler R package (53), and further ranking enrichment analysis was performed using the g: Profiler online tool (http://biit.cs.ut.ee/gprofiler/gost) (54). According to the importance of targets, the top 50 genes with the highest relevance to CRC were extracted (FDR < 0.05) to identify the more important biological entries and relevant pathways. The GOplot R package (55) was used to plot and visualize enrichment string maps.

### ROC and survival analysis of key exo-miRNAs

To obtain exo-miRNAs with high AUC, the pROC R package (56) was used for ROC analysis (the ROC curves of all SS and SC exo-miRNAs in Supplementary Figure 3). For combined-multivariate ROC analysis, logistic regression models were first developed using the glmnet R package (57), and further assessed by pROC.

Furthermore, to determine whether these exo-miRNAs and their target hotspot genes affect CRC patients' overall survival (OS), the survival R package (58) was used to conduct a survival analysis of key SC and SS exo-miRNAs. The TCGA survival data included both COAD and READ data, with means calculated for high and low expression groups, and log-rank *p* < 0.05 was considered a significant survival impact. GEPIA (59) was used to assess targeted hotspot genes for expression and survival analysis [367 CRC samples and 667 normal samples from TCGA (31) and GTEx (60) were included, |logFC| > 0.5, *p* < 0.05, survival analysis grouped by median expression].

### Response analysis of the EGFR-targeted drug for CRC cell lines

Twenty-two cell lines were grouped according to their median expression in the CCLE database (34). The sensitivity of two grouped cell lines to the EGFR-targeting drug lapatinib (as measured by IC50) was compared using a two-tailed *t*-test for each targeted EGFR tyrosine kinase inhibitor resistance pathway genes.

### ceRNA network construction and targeting pathway analysis of key exo-miRNAs

The following four steps were used to identify the key CRC exo-miRNA-associated ceRNA networks: (i) The lncRNAs positively associated with miRNA-targeted mRNA were extracted from cbioportal online tool (61) (640 COAD samples from TCGA; Spearman analysis, *p* < 0.05). (ii) The experimentally validated miRNA-lncRNA pairs were determined in the lncBase v2 database (62). (iii) The corresponding ceRNA was identified when lncRNAs and mRNAs linked to the same miRNA. (iv) The corresponding networks were built using Cytoscape (49). Targeted pathway analysis of 10 key miRNAs was accomplished using Cytoscape's ClueGO plugin (63), with relevant KEGG pathways selected at *p* < 0.05.

## Results

### Identification of differentially expressed serum exo-miRNAs in different stages of CRC patients

**Figure 3:**
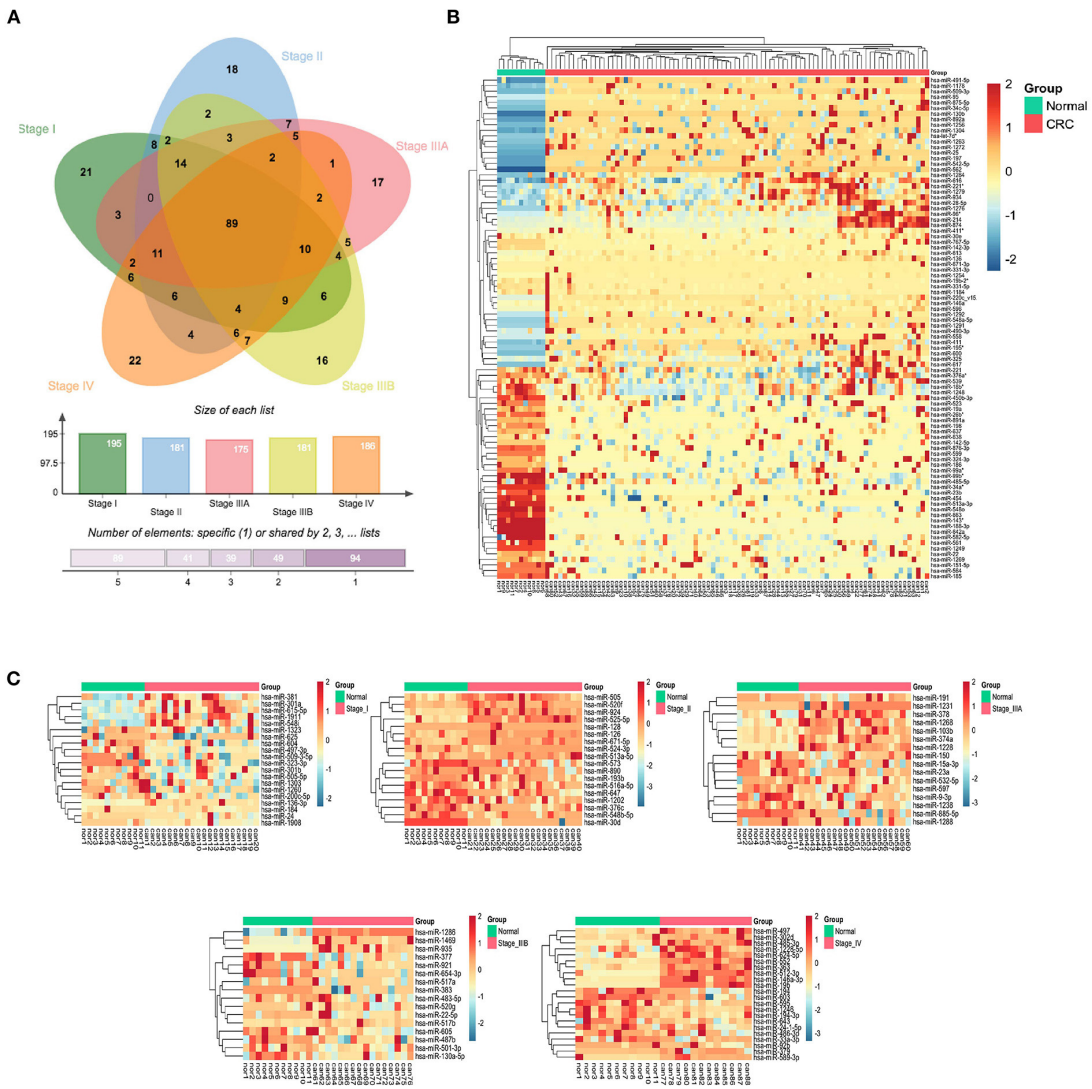
CRC stage common and specific exo-miRNAs. **(A)** Venn diagram of differentially expressed serum exo-miRNAs from 5 CRC stages. **(B)** Hierarchically clustered expression heat map of 89 differentially expressed serum SC exo-miRNAs. **(C)** Heat map of differentially expressed serum exo-miRNAs from 5 stages (I, II, IIIA, IIIB and IV).

Differential expression analysis of serum exo-miRNAs in different stages of CRC patients yielded 89 SC exo-miRNAs and 94 SS exo-miRNAs (p < 0.05, |logFC| > 1) (Figure 3A). Using hierarchical clustering of heatmaps, 89 SC exo-miRNAs were discovered to effectively distinguish CRC patients from healthy individuals (Figure 3B). The expression of SS exo-miRNAs was also investigated (Figure 3C), with stage I and IIIB being poorly differentiated, but stages II, IIIA, and IV being better differentiated, with stage IV showing the most significant difference.

### SC miRNA-target network construction of exo-miRNAs

**Figure 4:**
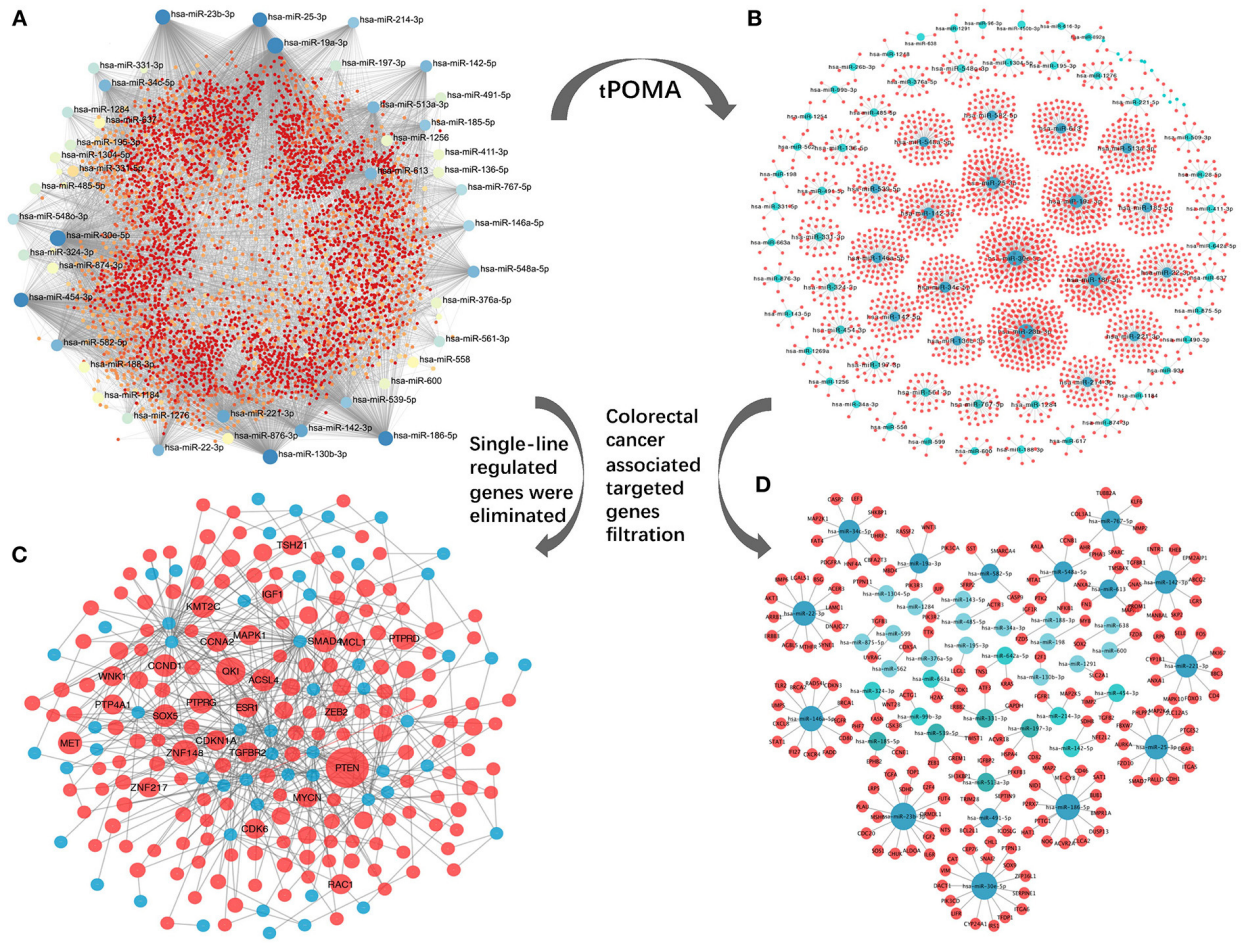
Construction of miRNA-target network. **(A)** Overall miRNA-target network of 89 CRC SC exo-miRNAs and targeting genes. **(B)** exo-miRNAs single-line regulatory network processed by tPOMA. **(C)** Hotspot gene network with single-line regulatory genes removed from the overall miRNA-target network and selected for CRC-related genes (all genes are regulated by at least two or more exo-miRNAs). **(D)** Genes associated with CRC screened from the single-line regulatory network to form the CRC-related gene network with single-line regulation of exo-miRNAs. Blue nodes represent corresponding exo-miRNAs, red nodes represent miRNA-regulated genes, larger nodes indicate more other nodes connecting to the node.

An exosomal miRNA-gene targeting network for the 89 SC exo-miRNAs was constructed (Figure 4A), which included 89 exo-miRNAs and their 7,306 predicted target genes from 11 databases (see Materials and methods section for detailed steps). Second, by establishing the tPOMA model (detailed steps are included in the Materials and methods section), a single-line regulatory network of exo-miRNAs was obtained in CRC (Figure 4B). Then, only genes with a high degree of relevance to CRC were selected from the overall miRNA-gene network and the miRNA single-line regulatory network (for detailed steps, see Materials and methods section). Concurrently, single-line regulated genes were excluded from the overall exosomal miRNA-gene network. Finally, a hotspot gene network was constructed including 56 exo-miRNAs and targeted 173 genes associated with CRC (at least two exo-miRNAs regulated each gene) (Figure 4C; Supplementary Table 2), as well as a miRNA single-line regulatory network constructed including 43 exo-miRNAs and targeted 186 genes associated with CRC (a single exo-miRNA regulated each gene) (Figure 4D; Supplementary Table 3).

### Exploring the biological function of genes with different targeting burden

To confirm that screened exo-miRNA-targeted genes are indeed related to the biological functions of exosomes and to compare the biological functions of exo-miRNA-regulated genes with varying targeting burdens, the corresponding enrichment analysis of biological functions was performed.

First, GO and KEGG enrichment analyses were performed for 173 hotspot genes and 186 single-line genes (Supplementary Figure 4A), respectively. It was found that these genes were enriched in signaling and regulation (e.g., signaling receptor binding, identical protein binding, enzyme binding, growth factor binding, and kinase activity); cell membrane complex structures (e.g., membrane-bounded organelle, membrane region, intracellular organelle, cell surface and receptor complex); regulation of substance secretion, transport and cellular response (e.g., positive regulation of metabolic process, positive regulation of cellular process, cellular response to chemical stimulus, positive regulation of biological process and cell population proliferation) and various biological pathways associated with cancer (e.g., colorectal cancer, gastric cancer, breast cancer, PI3K-AKT signaling pathway and proteoglycans in cancer). The findings show that these genes are important for secretory transport and exosome regulation.

**Figure 5:**
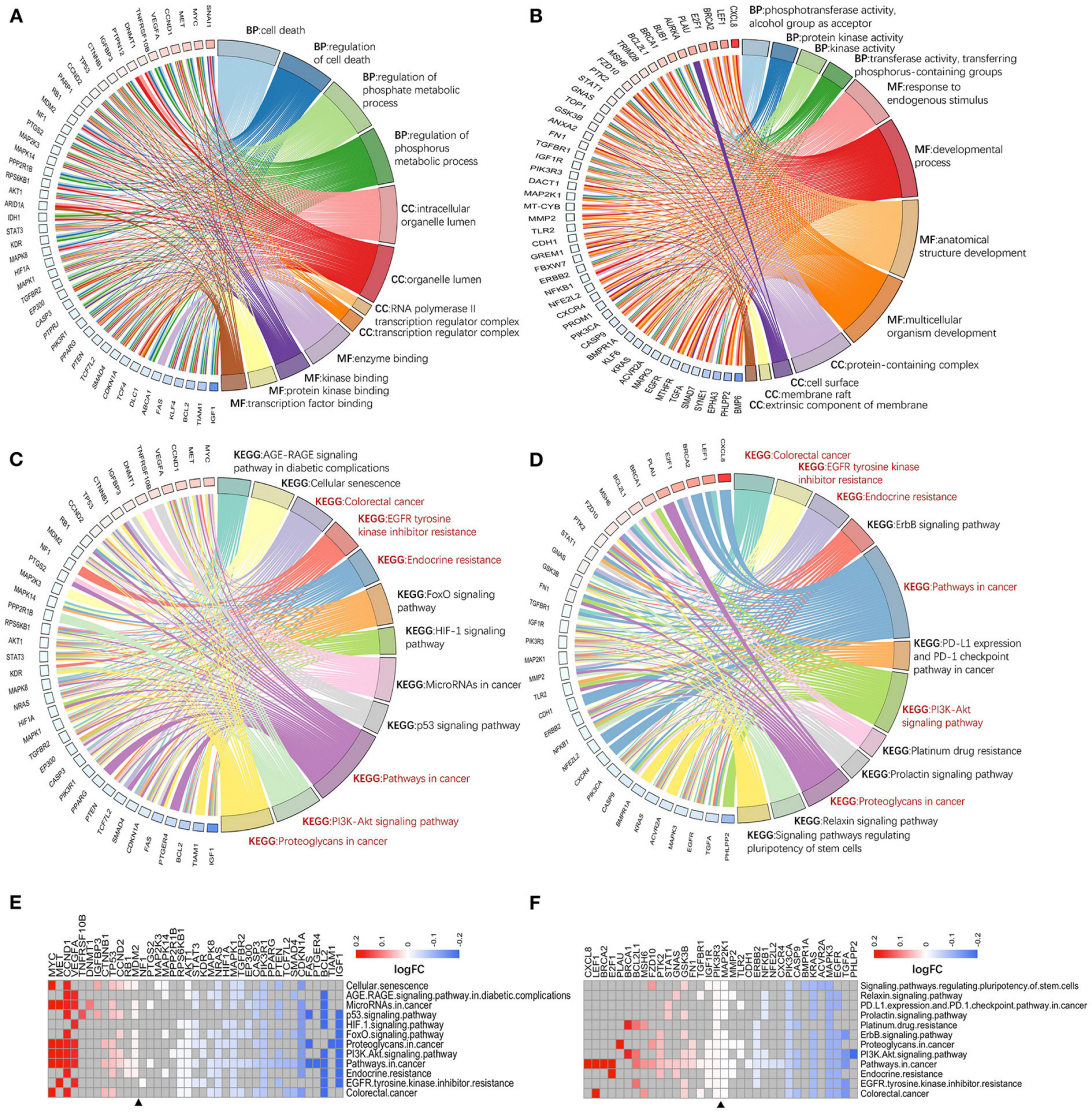
Enrichment analysis of genes with different targeting burdens. There is a difference between the entries of hotspot genes **(A)** and single-line regulated genes **(B)** in GO analysis. While the enriched pathways of hotspot genes **(C)** and single-line regulated genes **(D)** in KEGG have some intersection (red font), and each is enriched to different cancer-related pivotal pathways. Hotspot genes are usually down-regulated in these KEGG pathways **(E)**, and there is no significant difference between up-and down-regulation of single-line regulated genes in these pathways **(F)**. BP, biological process; MF, molecular function; CC, cellular component.

Second, two classes of genes was ranked based on CRC-related scores calculated by VarElect, and their biological functions were further explored using ranking enrichment analysis. Some differences in GO entries between hotspot genes (Figure 5A; Supplementary Table 4) and single-line regulated genes (Figure 5B; Supplementary Table 4) were discovered, as the former is more related to the regulation of cell death and phosphorylation metabolism in biological processes, with molecular functions focused on the binding of enzymes, proteins, and transcription factors, with the majority of these molecules localized in organelle membranes. The latter is primarily involved in activating enzymes in biological processes, molecular functions in cell development regulation and are primarily localized on the cell surface and membrane-related components. Both types of molecules are enriched in several cancer-associated pathways, including colorectal cancer, EGFR tyrosine kinase inhibitor resistance, endocrine resistance, PI3K/AKT signaling pathway, and cancer proteoglycans. However, they were also enriched in various pathways that are important for cancer development, immune checkpoint, drug tolerance, and pluripotent differentiation of stem cells (e.g., P53 signaling pathway, HIF-1 signaling pathway, FOXO signaling pathway, specific to hotspot genes; PD-L1 expression and PD-1 checkpoint pathway in cancer, platinum drug resistance, signaling pathways regulating pluripotency of stem cells, specific to single-line regulated genes) (Figures 5C,D; Supplementary Table 4).

Finally, the expression of genes from cancer-associated pathways was investigated. The hotspot genes being more down-regulated in these pathways (Figure 5E), which may be related to their negative regulation by multiple exo-miRNAs. In contrast, genes regulated by a single exo-miRNA were almost identical in terms of up-and down-regulation in these pathways (Figure 5F).

Through traditional and further ranking enrichment analysis, distinct regulatory patterns for two distinct types of genes with varying targeting burdens were identified in colorectal carcinogenesis and development, paving the way for future research into more precise regulation of exo-miRNAs.

### Identification of three key SC exo-miRNAs and five survival-related hotspot genes

**Figure 6:**
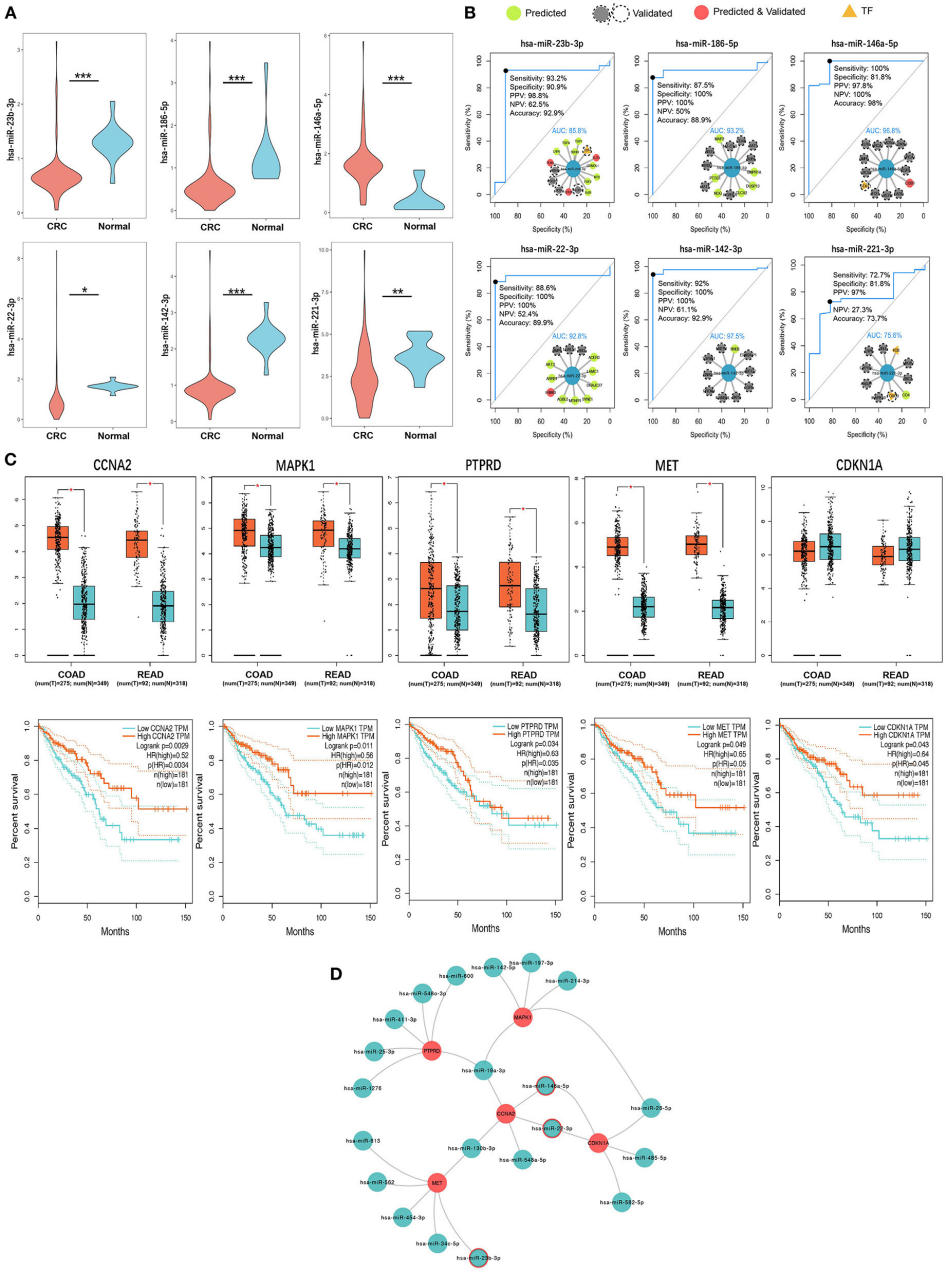
Six key CRC SC exo-miRNAs and five key survival-related hotspot genes were identified. **(A)** Six key miRNAs with significant expression differences in serum exosomes of CRC patients and healthy human serum exosomes. * *p* < 0.05; ** *p* < 0.01; *** *p* < 0.001. **(B)** ROC diagnostic performance assessment of the six key miRNAs (the internal inset shows this miRNA and its target CRC-related genes/TF; AUC, Area Under Curve). **(C)** Expression of five key hotspot genes with survival relevance in CRC and normal tissues (up) and corresponding Kaplan-Meier survival curves (down). **(D)** Five key hotspot genes targeted by 21 exo-miRNAs (miRNAs with a red border are both key single-line regulatory miRNAs and regulate hotspot genes together with other miRNAs).

According to the tPOMA model construction hypothesis, miRNAs targeting more CRC-related genes or transcription factors (TF) in the single-line regulatory network of exo-miRNAs may be more important. Additionally, in the hotspot gene network, CRC-related genes/TF targeted by a greater number of exo-miRNAs may be more susceptible to repressed. The corresponding exo-miRNAs and hotspot genes were arranged in descending order according to NCGSM-TF and INDEGREE (Table 1). To narrow down the screening of key miRNAs/genes and identify molecules of greater importance. NCGSM-TF and NCGSM were combined to thoroughly examine the top 15 miRNAs and hotspot genes with INDEGREE ≥ 5 (26 genes). Five of these miRNAs (miR-23b-3p, miR-186-5p, miR-22-3p, miR-142-3p and miR-221-3p) had significantly lower expression (Figure 6A) and high AUC (AUC > 85%, Figure 6B) in serum exosomes of CRC patients compared with healthy individuals, while miR-146a-5p demonstrated higher expression.

**Table 1:** Important miRNAs and hotspot genes screened based on miRNA-target network (top 26).

**miRNA**	**NCGSM-**	**NCGSM**	**NGSM**	**NGM**	**Gene**	**Indegree**
	**TF**	
miR-30e-5p	3	16	237	573	PTEN	13
miR-221-3p	2	10	75	251	QKI	8
miR-34c-5p	2	9	101	212	ZNF148	7
miR-767-5p	2	6	33	100	KMT2C	6
miR-539-5p	2	3	55	152	MET	6
miR-23b-3p	1	16	224	489	SMAD4	6
miR-146a-5p	1	13	87	126	CCND1	6
miR-25-3p	1	12	163	418	PTPRD	6
miR-548a-5p	1	6	78	210	PTPRG	6
miR-19a-3p	1	5	166	619	SOX5	6
miR-331-3p	1	3	30	73	ACSL4	6
miR-142-5p	1	2	66	204	TSHZ1	5
miR-186-5p	0	14	181	437	RAC1	5
miR-22-3p	0	12	78	182	WNK1	5
miR-142-3p	0	10	117	268	MAPK1	5
miR-613	0	4	82	206	CCNA2	5
miR-582-5p	0	4	64	195	ESR1	5
miR-491-5p	0	4	19	44	IGF1	5
miR-513a-3p	0	3	83	208	PTP4A1	5
miR-185-5p	0	3	68	152	TGFBR2	5
miR-197-3p	0	3	23	54	ZEB2	5
miR-214-3p	0	2	71	159	ZNF217	5
miR-324-3p	0	2	27	52	MYCN	5
miR-454-3p	0	2	26	415	CDKN1A	5
miR-642a-5p	0	2	8	17	CDK6	5
miR-663a	0	2	4	4	MCL1	5

NCGSM-TF, the number of CRC-related TF regulated in a single line by exo-miRNA; NCGSM, the number of CRC-related genes regulated in a single line by exo-miRNA; NGSM, the number of genes regulated in a single line by exo-miRNA; NGM, the number of genes regulated by exo-miRNAs; INDEGREE, the number of exo-miRNAs that regulate this gene.

Interestingly, most miRNAs in CRC patients' serum exosomes were down-regulated (data not shown), which is consistent with the general down-regulation of original miRNA levels in cancer patients (64) and suggests that most miRNAs in exosomes may act as cancer suppressors. Ten of 26 hotspot genes screened were upregulated in tissue expression in CRC patients, and seven were downregulated (|logFC| > 0.5, *p* < 0.05), and nine were below threshold (Supplementary Figure 5). Furthermore, five genes (*CCNA2, MAPK1, PTPRD, MET*, and *CDKN1A*) were significantly associated with CRC patients' better survival (Figure 6C). There is reason to believe that these genes with a high miRNA suppressive burden in CRC patients act as protective molecules in the compensatory increase after tumor occurrence and positively correlate with patient survival. Finally, the exo-miRNA regulatory network of five survival-related genes was constructed (Figure 6D). Three miRNAs were identified (miR-146a-5p, miR-22-3p, and miR-23b-3p) with the strong single-line regulatory ability and the ability to regulate hotspot genes in conjunction with other exo-miRNAs.

### Construction of miRNA-target network and subnetworks for SS exo-miRNAs

**Figure 7:**
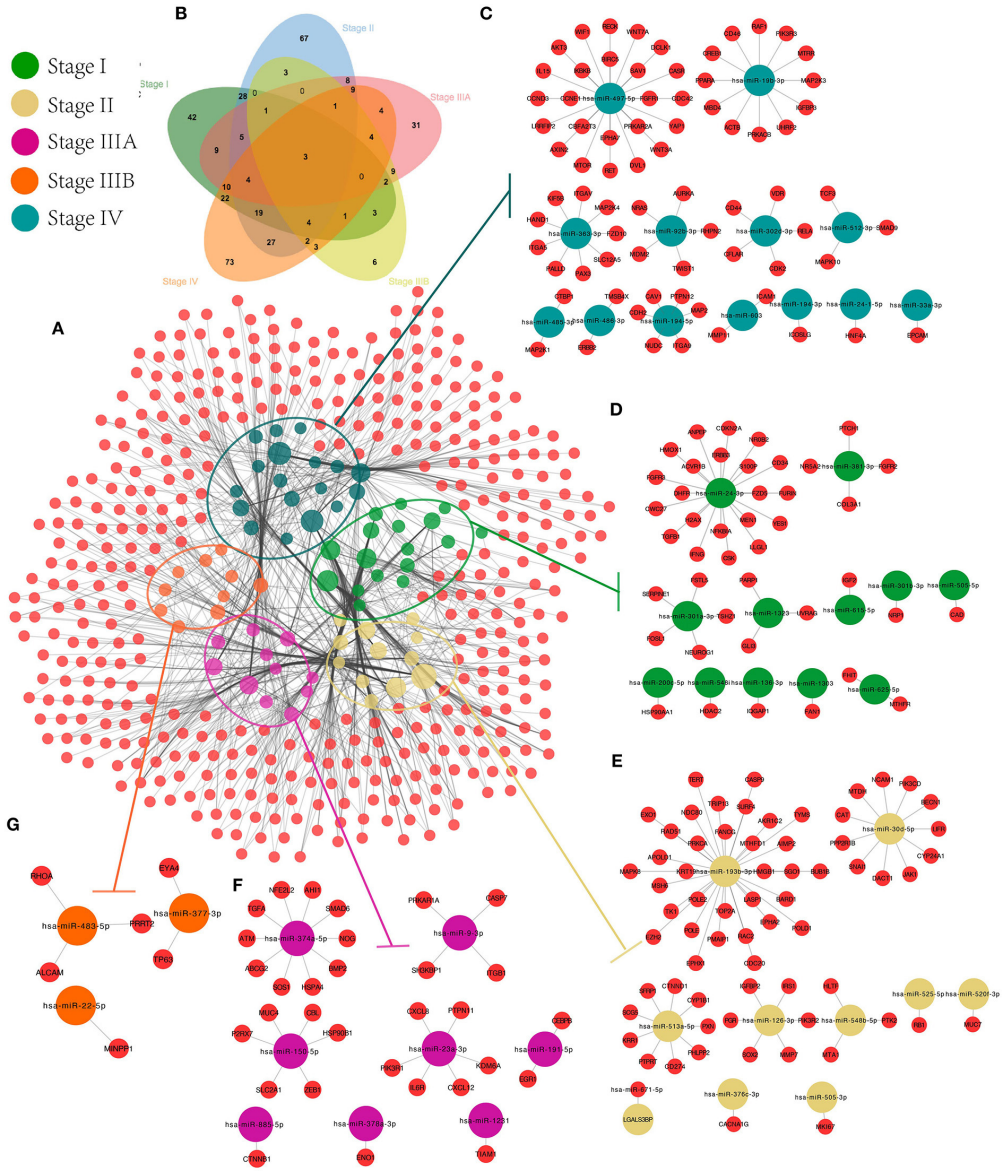
CRC exo-miRNAs SS network construction. We constructed the overall miRNA-target network for 94 SS exo-miRNAs **(A)**, obtained the target genes specific to each stage by Venn diagram **(B)**, and extracted the SS miRNA-target subnetworks from the overall miRNA-target network: IV **(C)**, I **(D)**, II **(E)**, IIIA **(F)**, and IIIB **(G)**.

First, target genes and screened CRC-related genes were identified for all SS exo-miRNAs obtained based on 11 databases, and an SS miRNA-target network was constructed (Figure 7A). Subsequently, the CRC-related genes of each stage were identified by Venn diagram (Figure 7B), and finally, five SS CRC miRNA-target sub-networks were obtained (stage IV, Figure 7C; stage I, Figure 7D; stage II, Figure 7E; stage IIIA, Figure 7F; stage IIIB, Figure 7G). Additionally, GO and KEGG enrichment analyses were performed to explore the role of different SS subnetworks in CRC development.

According to the results of GO analysis, stage I focuses on cellular development and metabolic processes (e.g., cellular development process, cell differentiation, and positive regulation of metabolic process) and is found on the cell surface, endomembrane system, vesicle, and intracellular vesicle (Supplementary Figure 4B). Stages II and IIIA have increased signal transduction and response (e.g., regulation of cell communication, signaling, signal transduction, and chemical response). They are associated with transcriptional regulation and cellular junctions (e.g., transferase complex, chromosome, DNA polymerase complex, and cell-cell junction). The main focus of stage IV is on metabolic and phosphorylation-related processes (e.g., positive regulation of the cellular metabolic process, phosphorus metabolic process, and phosphorylation regulation), which are closely related to cell adhesion and transcriptional regulation (e.g., cell junction, anchoring junction, focal adhesion, and transcription regulator complex). All stages are linked to molecular binding and enzyme activation in molecular function. Furthermore, genes in different SS subnetworks showed some pathway similarities and differences.

The effects of exo-miRNAs-regulated genes on CRC progression and changes in biological processes were observed by exploring the biological functions of genes in different subnetworks, which aids in understanding the regulation of exo-miRNAs in different stages of CRC patients.

### Identification of seven key SS exo-miRNAs

The expression and ROC curve diagnostic performance of all SS exo-miRNAs were evaluated. There were two I-specific miRNAs (miR-301a-3p, miR-548i), two IIIA-specific miRNAs (miR-23a-3p, miR-532-5p), and six IV-specific miRNAs (miR-1246, miR-194-3p, miR-33a-3p, miR-485-3p, miR-194-5p and miR-379-5p) present high Stage-AUC (Stage-AUC > 65%, it represent the performance of miRNA can distinguish one stage from other stages).

**Figure 8:**
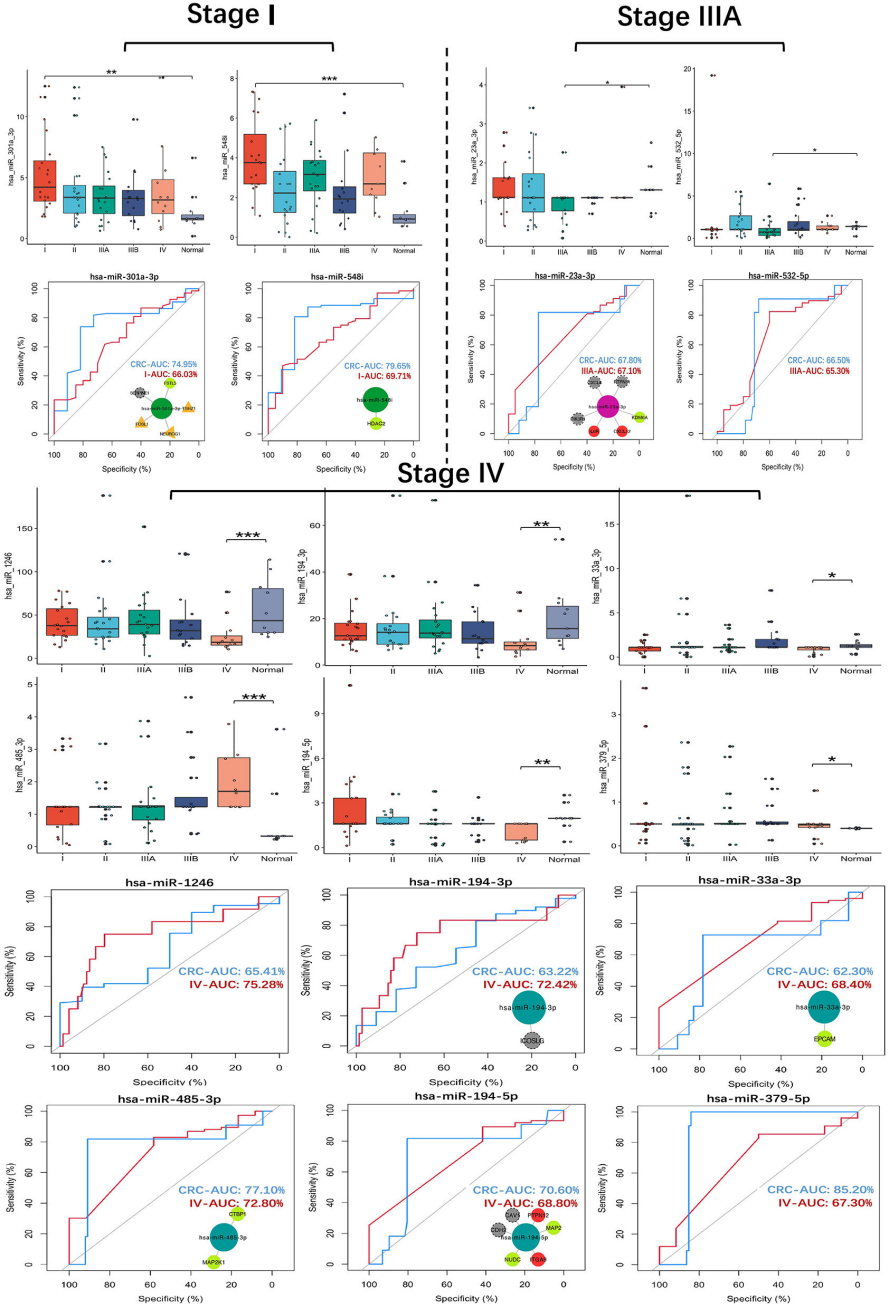
SS exo-miRNAs expression assessment and ROC curve analysis. ^*^p < 0.05;^**^p < 0.01;^***^p < 0.001. CRC-AUC: area under the curve of this miRNA in CRC patients and healthy human serum exosomes; Stage-AUC: area under the curve of this miRNA in this stage versus other stages; ROC curves with network insets are key miRNAs and their regulated SS CRC-related genes present in the SS subnetworks.

**Figure 9:**
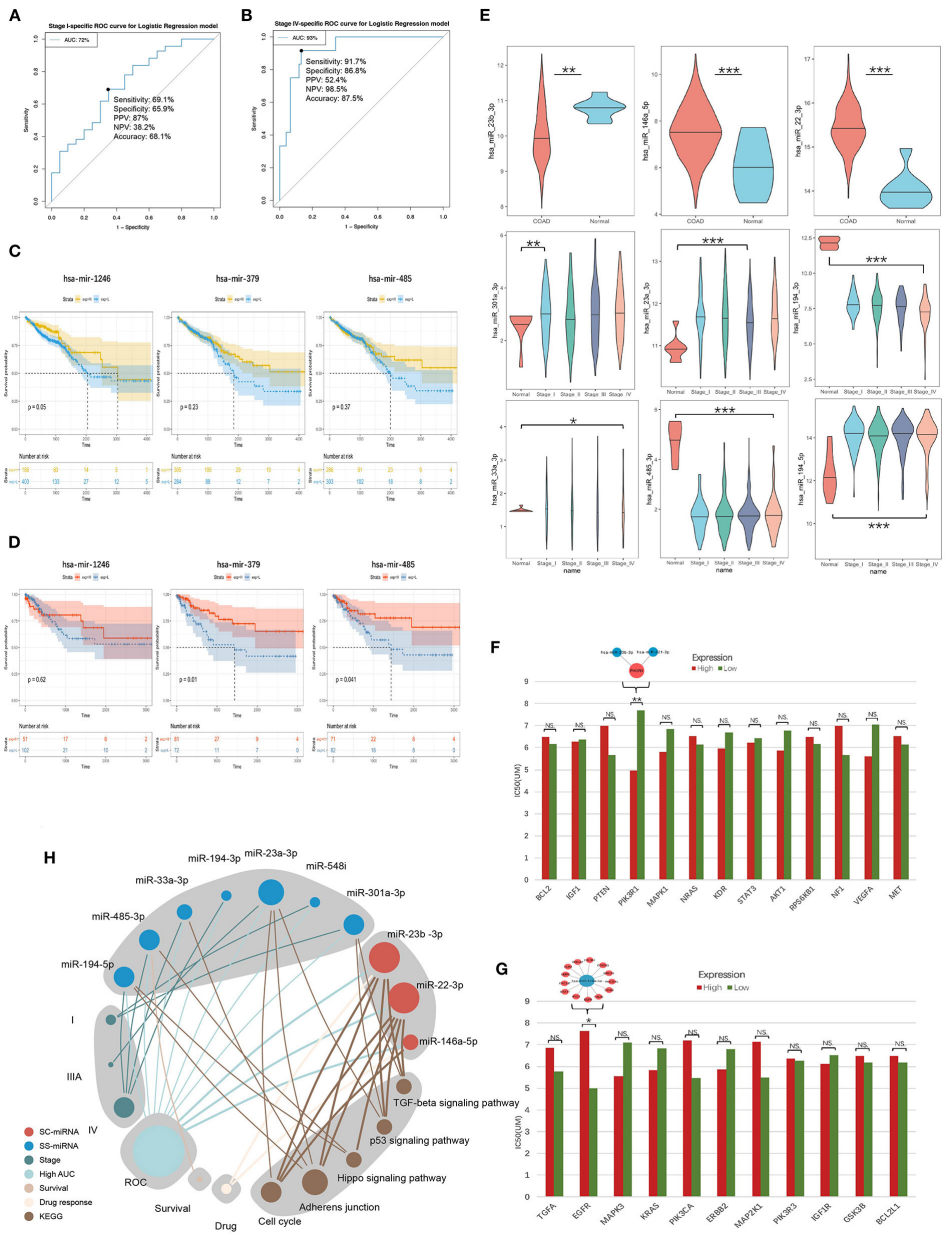
Survival analysis and drug response analysis. **(A)** ROC curves include two stage I-specific exo-miRNAs (miR-301a-3p, miR-548i) for combined differentiation of stage I and other stages of CRC patients. **(B)** ROC curves include four stage IV-specific exo-miRNAs (miR-194-3p, miR-33a-3p, miR-485-3p and miR-194-5p) were used to jointly distinguish stage IV CRC patients from other stages. **(C,D)** Kaplan-Meier curve demonstrated mir-1246 was associated with overall survival, mir-379 and mir-485 were associated with 3-years later survival of CRC patients. **(E)** Tissue expression assessment of three key SC exo-miRNAs and six SS exo-miRNAs in CRC (*p < 0.05; ** *p* < 0.01; ***p < 0.001). **(F)** EGFR tyrosine kinase inhibitor resistance analysis of hotspot genes in the EGFR tyrosine kinase inhibitor tolerance pathway (the higher the IC50 value, the stronger the resistance; * *p* < 0.05; ** *p* < 0.01; *** *p* < 0.001). **(G)** Resistance analysis of single-line regulated genes in the EGFR tyrosine kinase inhibitor tolerance pathway (*p < 0.05; ** *p* < 0.01; *** *p* < 0.001). **(H)** Summary of molecular features of 10 key exo-miRNAs.

Their stage diagnostic performance (Stage-AUC) was 65–75%, and their overall diagnostic performance (the miRNA can distinguish CRC patients from healthy people) was 62–85% (Figure 8; Supplementary Table 5). Furthermore, seven miRNAs found in previously constructed SS subnetworks were selected as key SS exo-miRNAs, with two for stage I (miR-301a-3p and miR-548i), one for stage IIIA (miR-23a-3p), and four for stage IV (miR-194-3p, 33a-3p, miR-485-3p and miR-194-5p). Given the low stage differentiation properties of these SS exo-miRNAs, a combined diagnostic analysis of two I-specific exo-miRNAs and four IV-specific exo-miRNAs was performed, with the stage I combined diagnostic model achieving 72% AUC (Figure 9A) and the stage IV combined diagnostic model achieving 93% AUC (Figure 9B).

### Survival and expression validation of SC/SS miRNA

A survival analysis based on CRC data in TCGA was performed to further understand the impact of these SC/SS exo-miRNAs on the survival of CRC patients. It was discovered that low expression of mir-1246 was associated with worse overall survival (OS) of patients (Figure 9C), while low expression of mir-379 and mir-485 predicted worse survival after 3 years (Figure 9D). Additionally, the expression of key exo-miRNAs in colon cancer tissues and healthy tissues were explored in TCGA (Figure 9E), in which the miR-23b-3p, miR-146a-5p, miR-301a-3p, and miR-194-3p present the same expression changes compared with CRC serum exosomes. The miRNA expression was further validated in GSE115513, and most of the miRNA expression alterations were similar to that of the TCGA cohort, except for miR-194-5p (SS-IV) had the opposite expression (Supplementary Figure 6). Among them, both tissue and exosome expression of miR-194-3p (SS-IV) were significantly decreased in stage IV, suggesting its possible role as a potential tumor suppressor miRNA for exosomal transport. Finally, the expression of key exo-miRNAs in the exosomes of five different CRC cell lines was investigated (Supplementary Figure 7). The expression of exo-miRNAs in CRC cell line exosomes was largely consistent with that in serum exosomes (e.g., miR-22-3p, miR-23b-3p, miR-301a-3p, miR-194-3p, miR-485-3p). In summary, miR-23b-3p, miR-301a-3p and miR-194-3p were validated being the most stably expressed stage-associated miRNAs in CRC serum exosomes, cell exosomes and tissues (Table 2).

**Table 2:** Expression changes of key miRNAs in different datasets.

**Type**	**miRNA**	**Serum**	**Cell**	**Tissue**	**Tissue**
		**exosome**	**exosome**
		GSE39833	GSE39832	TCGA	GSE115513
			GSE39814		
SC	miR-146a-5p	Up	Up/down	Up	Up/down
	miR-22-3p	Down	Up/down	Up	Up/down
	miR-23b-3p	Down	Down	Down	Down
SS-I	miR-301a-3p	Up	Up	Up	NA
	miR-548i	Up	Up/down	NA	NA
SS-IIIA	miR-23a-3p	Down	Up/down	Up	Up
SS-IV	miR-194-3p	Down	Down	Down	Down
	miR-33a-3p	Down	Up	Down	NA
	miR-485-3p	Up	Up/down	Down	NA
	miR-194-5p	Down	Up/down	Up	Down

### Identification of exo-miRNAs and their target genes that respond to the EGFR-targeting drug

The EGFR tyrosine kinase inhibitor tolerance pathway was enriched in hotspot genes and single-line regulated genes. The response of exo-miRNAs regulatory genes in this pathway to the EGFR-targeted drug lapatinib was analyzed to further identify exo-miRNAs regulatory genes with tolerance response to EGFR tyrosine kinase inhibitors (of the three EGFR targeting drugs in the CCLE database, only lapatinib responded to these genes). Low PIK3R1 expression among the hotspot genes in the CRC Cell significantly increased resistance to lapatinib (Figure 9F), and the previously identified key SC exo-miRNAs: miR-23b-3p and miR-221-3p were able to regulate this gene directly. In contrast, only high EGFR expression among single-line regulated genes in the CRC cell increased resistance to lapatinib (Figure 9G), and EGFR was directly regulated by miR-146a-5p.

Finally, the potential pathways, ROC diagnostic capabilities, survival, and drug response of 10 key stage-associated exo-miRNAs were summarized to demonstrate the potential importance of each miRNA (Figure 9H). MiR-22-3p, miR-23a-3p and miR-23b-3p are the top3 important stage-associated exo-miRNAs, and they display close correlation with several vital cancer-associated pathways such as TGF-beta signaling, p53 signaling, and hippo signaling pathways.

### Exploring the potential ceRNA regulatory functions of 10 key exo-miRNAs

**Figure 10:**
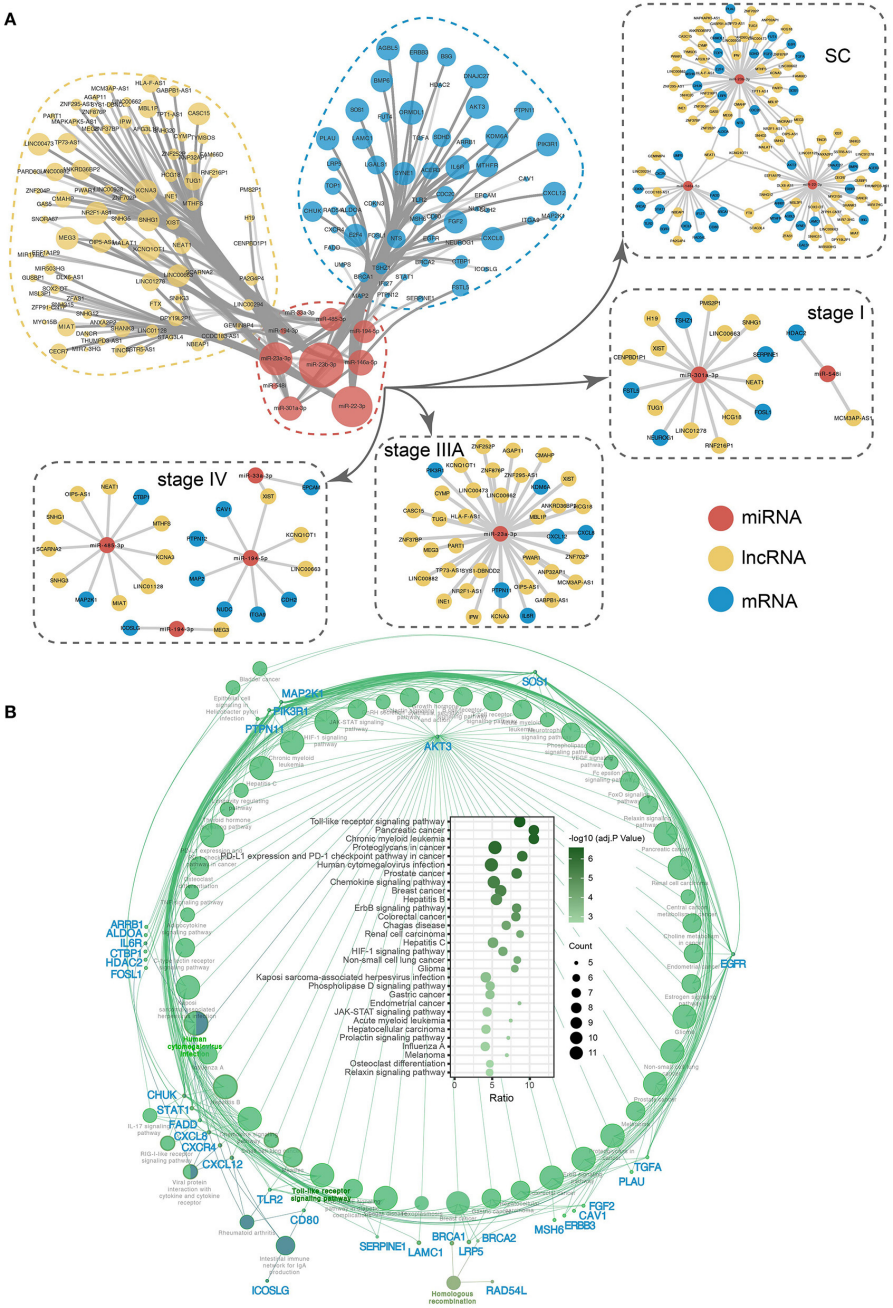
The ceRNA network and KEGG pathway of 10 key exo-miRNAs. **(A)** The ceRNA network and subnetwork of SC and SS exo-miRNA. **(B)** The miRNA targets-pathway network displaying the correlation between stage-associated exo-miRNA targets and pathways. Bubble plot depicting significantly enriched pathways.

In addition to targeting the corresponding mRNAs, inhibiting their expression, and influencing downstream functions, miRNAs can also interact with the corresponding lncRNAs, and a competitive relationship between lncRNAs and mRNAs that bind to the same miRNA, known as competing endogenous RNA (ceRNA) regulation, can form. To explore the potential ceRNA regulation of stage-associated exo-miRNAs, the ceRNA network was constructed (Figure 10A; Supplementary Table 6). NEAT1 and KCNQ1OT1 are lncRNAs associated with all three ceRNA regulatory modules in the SC exo-miRNA ceRNA network. NEAT1 and KCNQ1OT1 have been reported to be upregulated in a variety of cancers and promote tumorigenesis by altering the expression levels of sponged miRNAs (65, 66). Additionally, the corresponding ceRNA regulatory networks in stages I, IIIA, and IV were identified, which have corresponding roles in regulating the various stages. To better understand the impact of these miRNAs on downstream biological pathways, their miRNA targeting pathways were analyzed using the ClueGO plugin in Cytoscape (63). There were 59 significantly enriched pathways (Supplementary Table 7), among which Toll-like receptor signaling pathway is the most significant (adj. *p* = 2.60e−07, adjusted by Bonferroni step down) (Figure 10B). Additionally, *AKT3* was identified as the gene that is most strongly associated with all pathways and is involved in a variety of cancer-related processes. *AKT3* is a member of the protein encoding gene of AKT serine-Threonine protein kinase. AKT is usually overexpressed in CRC and serves as a key target of targeted therapy based on PI3K/AKT signaling pathway (67, 68).

## Discussion

Identification of stage-associated exo-miRNAs biomarker is critical for better understanding the unique developmental progression of CRC at different stages, developing optimal stage monitoring protocols, and determining precise tumor treatment strategies. However, traditional miRNA markers are frequently discovered based on their altered expression and discriminatory ability between experimental and control groups, ignoring their regulatory ability and biological function. Combining the differential expression alteration and *in vivo* targeting or the regulatory ability of miRNA target molecules with their function and disease relevance can aid in identifying more reliable miRNA biomarkers.

The miRNA targeting network considers miRNAs' targeting and regulatory abilities and effectively identify disease-related miRNAs. However, a single miRNA can frequently regulate the expression of a large number of genes simultaneously, and the diversity of regulatory genes frequently complicates our understanding of the miRNA's regulatory function. The POMA model developed by Chen et al. (24) simplifies miRNA regulation by identifying single-line regulatory molecules and vulnerable nodes in the miRNA regulatory network. These genes, which are only regulated by one miRNA, avoid regulatory interference from other miRNAs, effectively reflecting that miRNA's regulatory specificity. This concept was applied to screening key exo-miRNAs in this study, resulting in developing a screening strategy for the tPOMA. Finally, 10 stage-associated exo-miRNAs were identified by tPOMA and further stage subnetwork analysis, ROC analysis, survival analysis and drug response analysis.

Three key SC exo-miRNAs (miR-146a-5p, miR-22-3p, and miR-23b-3p), which can significantly discriminate CRC patients and healthy individuals (AUC = 96.8, 92.8, and 85.8%). miR-146a-5p expression is significantly increased in CRC tissues and promotes CRC cell migration (69), which likely explains the significant increase in miR-146a-5p expression in CRC serum exosomes. Similarly, the significant increase of miR-146a-5p in exosomes may promote CRC cell migration, which needs to be further investigated. And miR-146a-5p also induces tumor formation by inhibiting NUMB protein, resulting in the asymmetric segregation of CRC stem cells (70). The miR-22-3p was found to be significantly upregulated in CRC tissues (71), but it was downregulated in serum and cell exosomes from our results. Sastre et al. (72) transfected miR-22-3p mimics into CRC cells (HCT116), which were found to inhibit cell proliferation and induce cell death significantly. However, miR-23b-3p was downregulated in CRC tissues, serum exosomes, and cell exosomes, which has been identified as a key regulatory molecule in some cancers (73–77). Ostenfeld et al. (78) discovered that miR-23b-3p was elevated in extracellular vesicles of CRC blood samples and decreased after the tumor was surgically removed. It appears to be different from our finding of reduced miR-23b-3p in CRC patients' serum exosomes, which may be caused by the tumor heterogeneity.

Seven key SS exo-miRNAs include two key I-specific exo-miRNAs (miR-301a-3p and miR-548i), one key IIIA-specific exo-miRNA (miR-23a-3p) and four key IV-specific exo-miRNAs (miR-194-3p, miR-33a-3p, miR-485-3p, and miR-194-5p). The miR-301a-3p is significantly upregulated in CRC tissues (79, 80), and Zhang et al. (80) discovered that overexpression of miR-301a-3p promoted CRC cell proliferation, migration, invasion, as well as the growth of xenograft tumors and liver metastasis *in vivo*. However, miR-548i was found to be significantly downregulated in early colon adenocarcinoma tissue when compared to precancerous and colonic intraepithelial neoplasia tissue (81). Additionally, miR-301a-3p and miR-548i were both upregulated in CRC patients' serum exosomes and were most abundant in stage I patients, suggesting a role for early detection of CRC. Along with miR-376c-3p, miR-23a-3p has been successfully used as a serum marker for colorectal cancer (CRC) to predict 3-year OS in 70% of patients (82). Notably, it was discovered that mir-485 in CRC-IV exosomes could predict patients' better survival after 3 years at the level of CRC tissues, which may aid in the prognosis of advanced CRC patients. In CRC-IV exosomes, miR-194-3p and miR-194-5p showed intriguing results. miR-194-3p expression was significantly downregulated in CRC tissues, whereas miR-194-5p expression was significantly upregulated, but both were significantly downregulated in exosomes. According to Zhang et al. (83), CRC cells' migration and invasion ability transfected with mir-194 mimics appeared to be reduced. Still, it is unknown whether CRC cells can enhance their migration and invasion ability by decreasing miR-194 family secretion in exosomes.

Finally, it was discovered that a drug-resistance pathway (EGFR tyrosine kinase inhibitor resistance) was enriched for both types of targeted genes and that CRC cell lines with low expression of *PIK3R1* (targeted by exosome miR-23b-3p/miR-221-3p) exhibited resistance to the EGFR-targeted drug lapatinib. Additionally, CRC cell lines with high *EGFR* (targeted by exosome miR-146a-5p) also demonstrated resistance to lapatinib. Briefly, the reduced expression of SC miR-23b-3p and elevated expression of miR-146a-5p in serum exosomes of CRC patients (Figure 6A) may help to enhance the sensitivity to EGFR-targeted drugs in all patients without considering stage. Concurrently, SS-IIIA miR-23a-3p could also target *PIK3R1* (Figure 7F), the expression of miR-23a-3p was specifically reduced in serum exosome of stage IIIA CRC patients, which may help to enhance the response to EGFR-targeted drugs in stage IIIA patients. The regulation of some CRC-related genes by these exo-miRNAs exhibited distinct responses to drug treatment, suggesting that exo-miRNAs may represent hope for drug-resistant therapy in some CRC patients. Moreover, the genes targeted by stage-associated exosomal miRNA are indeed involved in many main processes of colorectal carcinogenesis and important cancer-related pathways such as: angiogenesis, immune regulation, cellular metabolism (Supplementary Table 4), and TGF-beta, p53, hippo signaling pathways (Figure 9H). In detail, we found that miR-23a-3p (SS-IIIA) could target *CXCL8* (Figure 7F), which has been reported in several studies to play a key role in angiogenesis in CRC (84), and the specific reduction of miR-23a-3p in exosomes from stage IIIA patients may upregulate *CXCL8* expression and thus promote angiogenesis. In addition, inflammatory response is one of the typical features of CRC, and *SERPINE1* is one of the targets of miR-301a-3p (SS-I) (Figure 7D), which has been reported to play a key role in shaping the immune microenvironment and activation of immune-related pathways in CRC (85).

In general, these stage-associated exo-miRNAs have been studied in tissue/cell from CRC or other cancers, but they have been reported in exosomes relatively infrequently or not at all. Despite some sample type differences, a corresponding comparison and side-by-side validation were performed using cellular exosomes and tissues data. A literature mining-based confirmatory survey of the 10 CRC exosome-associated miRNAs identified as markers was also performed further to corroborate our study. Finally, 159 studies had been investigated and the pro-/anti-tumor effects of 10 stage-associated exo-miRNAs were revealed (Supplementary Table 8). It is worth noting that there are still some limitations of the present study. First, the main focus of our study was on the functional impact of these stage-related exo-miRNAs on CRC, and the limited amount of data does not support their use as valid diagnostic indicators. Second, although we obtained three exo-miRNAs stably expressed in cellular exosomes, serum exosomes and tissues, their pro-/anti-tumor effects need further experimental validation.

## Conclusion

Three SC and seven SS exo-miRNAs were identified by stage-related miRNA-target network analysis and assessed by ROC analysis. Three exo-miRNAs were validated being stably expressed in serum exosomes, cell exosomes and tissues. These stage-associated exo-miRNAs are expected to be new biomarkers for CRC patients' staging surveillance and further mechanism research.

## Data availability statement

The datasets presented in this study can be found in online repositories. The names of the repository/repositories and accession number(s) can be found in the article/Supplementary material.

## Author contributions

FL and LT designed the ideas for this paper. ZC, YT, and JL contributed to data collation and data analysis. FL analyzed and interpreted the data. All authors contributed to the writing of the manuscript. All authors contributed to this article and approved the submitted version.

## Funding

This work was funded by the Science Innovation Program of College of Laboratory Medicine, Chongqing Medical University (CX201704).

## Conflict of interest

The authors declare that the research was conducted in the absence of any commercial or financial relationships that could be construed as a potential conflict of interest.

## Publisher's note

All claims expressed in this article are solely those of the authors and do not necessarily represent those of their affiliated organizations, or those of the publisher, the editors and the reviewers. Any product that may be evaluated in this article, or claim that may be made by its manufacturer, is not guaranteed or endorsed by the publisher.

## Supplementary material

Supplementary Figure S1 PCA quality control and volcano plot of exo-miRNAs expression in five CRC stages versus healthy individuals.

Supplementary Figure S2 Reliability verification of tPOMA model.

Supplementary Figure S3 ROC and expression of all stage-specific exo-miRNAs.

Supplementary Figure S4 Enrichment analysis of stage-common and stage-specific exo-miRNAs target genes.

Supplementary Figure S5 Expression of 26 hotspot genes in COAD and READ tissues.

Supplementary Figure S6 Tissue expression validation of three key SC exo-miRNAs and seven SS exo-miRNAs in GSE115513.

Supplementary Figure S7 Expression of 10 key exo-miRNAs in exosomes of five CRC cell lines.

Supplementary Table 1 CRC exosome datasets collection results.

Supplementary Table 2 Hotspot gene network and CRC-related scores.

Supplementary Table 3 Single-line regulated genes and CRC-related score.

Supplementary Table 4 Results of GO and KEGG enrichment analysis for hotspot genes and single-line regulated genes.

Supplementary Table 5 Sensitivity, specificity, PPV, NPV and accuracy of SS exo-miRNAs.

Supplementary Table 6 ceRNA.

Supplementary Table 7 Results of ClueGO enrichment analysis for 10 key exo-miRNAs.

Supplementary Table 8 Literature verification of 10 key exo-miRNAs.
