# Supplementary material for: Identification of stage-associated exosome miRNAs in colorectal cancer by improved robust and corroborative approach embedded miRNA-target network
Source: Front Med (Lausanne). 2022 Sep 27;9:881788. doi: 10.3389/fmed.2022.881788 (PMC9551196; doi:10.3389/fmed.2022.881788)
Supplement: Supplementary Figure S1 — PCA quality control and volcano plot of exo-miRNAs expression in five CRC stages versus healthy individuals. [file Data_Sheet_1.DOCX]

Supplementary Material


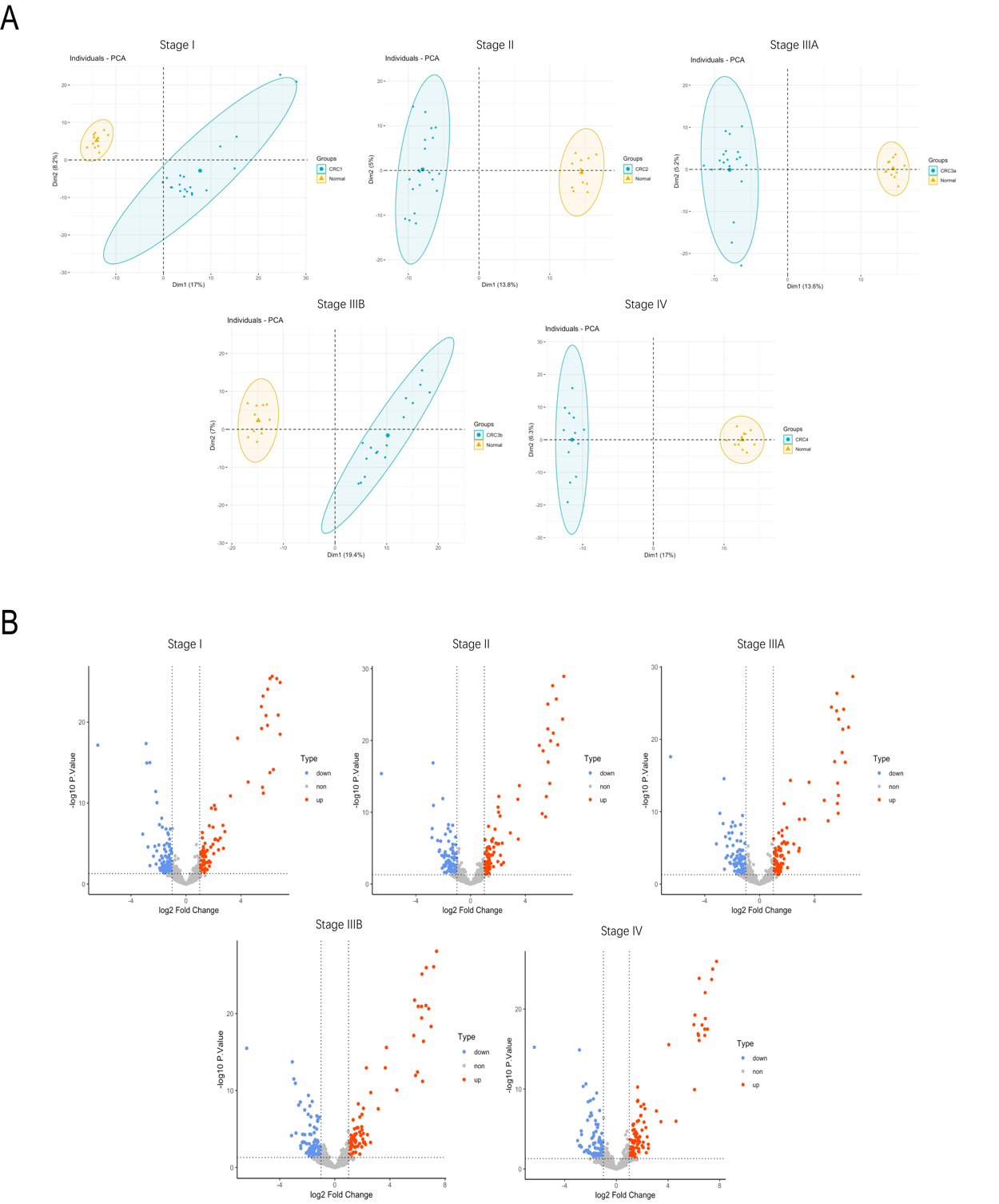


**Supplementary Figure 1| PCA quality control and volcano plot of exo-miRNAs expression in five CRC stages versus healthy individuals. A:** PCA plots of serum exosomal miRNA expression data from patients with five CRC stages versus healthy individuals, showing that the five stages can be significantly distinguished from healthy individuals. **B:** Volcano plots of differential analysis of serum exosomal miRNA expression from patients with five CRC stages. Red represents upregulated miRNAs, blue represents downregulated miRNAs. p < 0.05, |logFC| > 1.


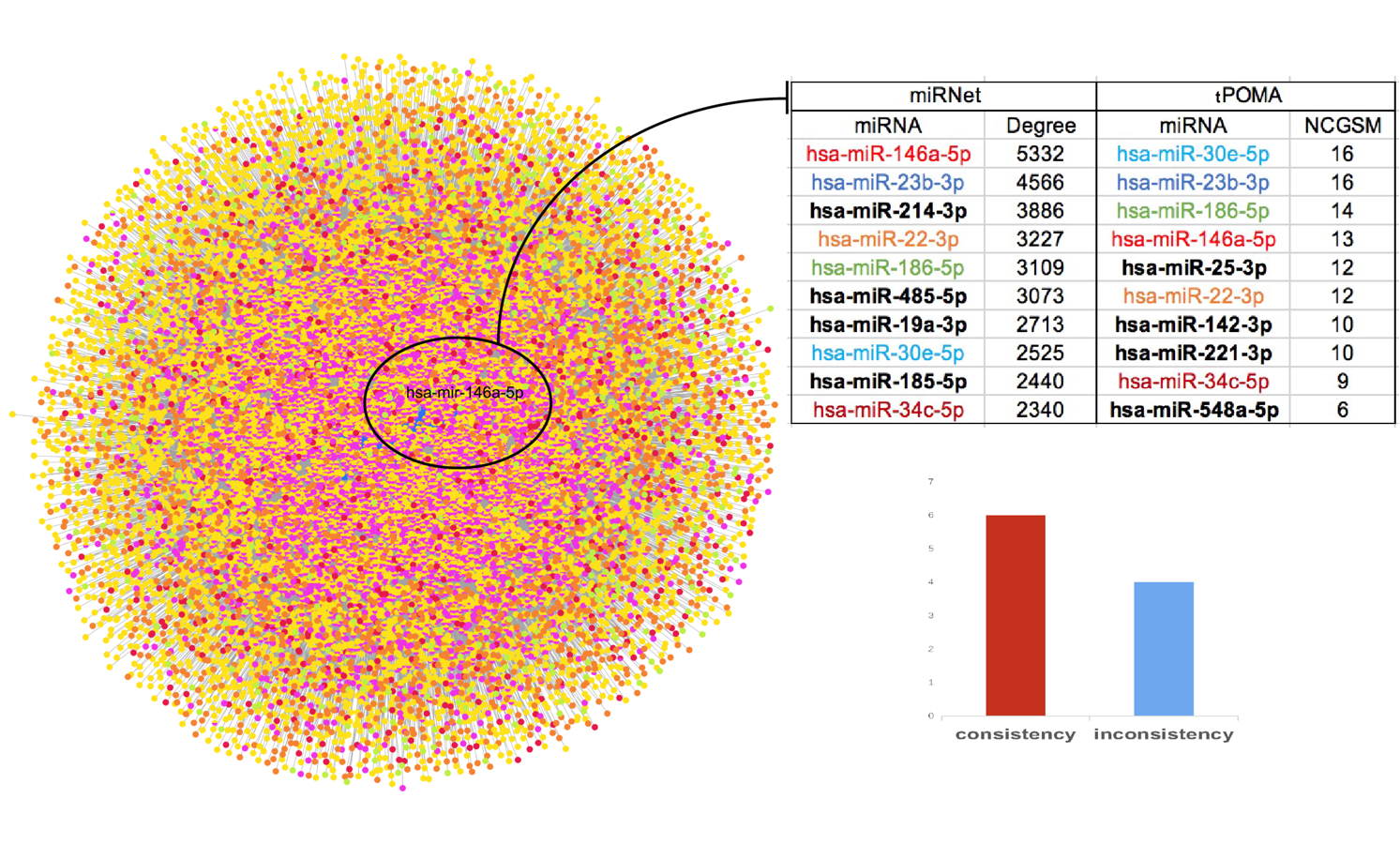


**Supplementary Figure 2| Reliability verification of tPOMA model.** 89 SC exo-miRNAs targeting network constructed by miRNet and comparison between miRNet and sPOMA. The top 10 miRNAs screened by sPOMA model had high consistency (60%) with miRNet (colored in the table are same miRNAs, bolded in black are inconsistent miRNAs).


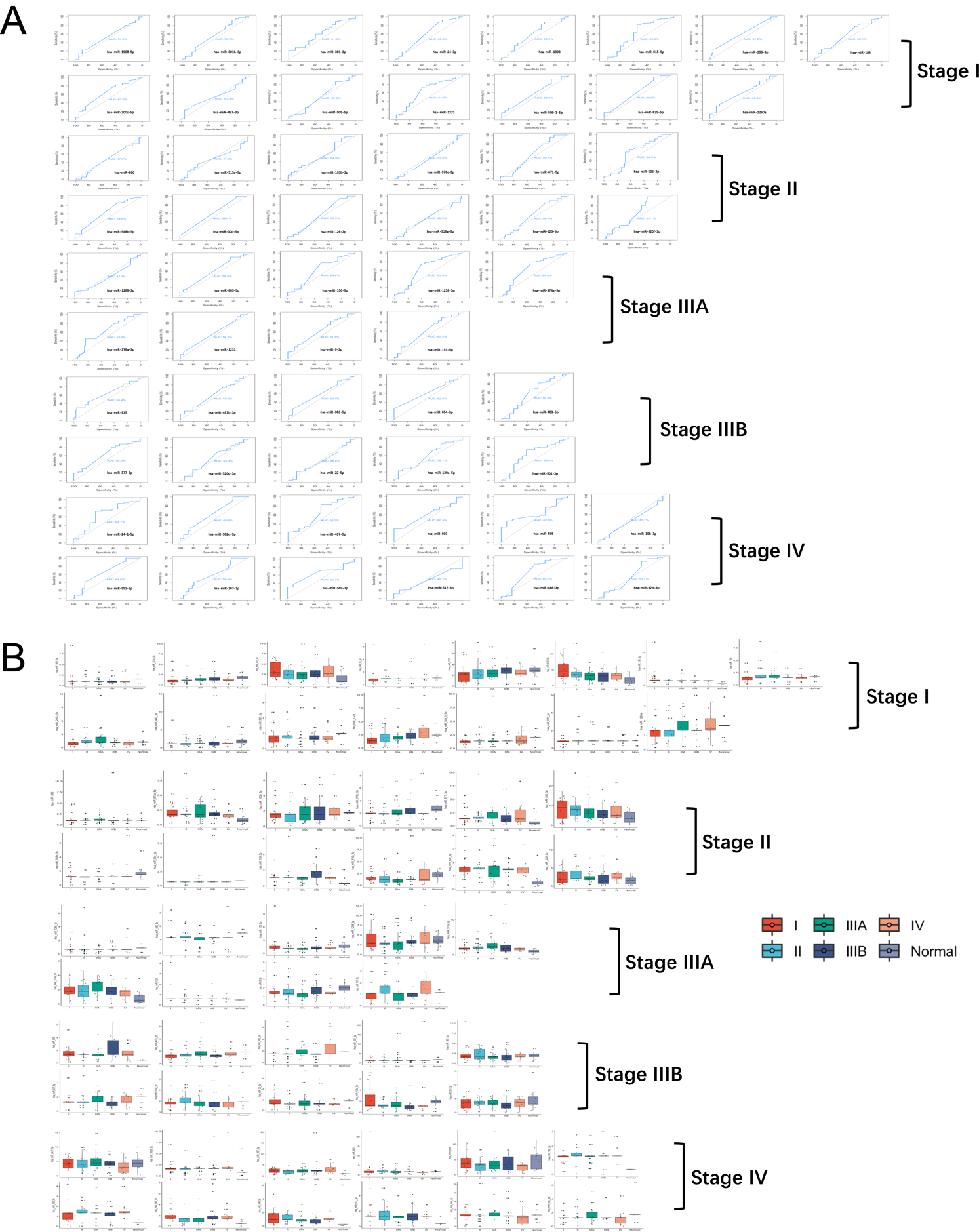


**Supplementary Figure 3|** **ROC and expression of all stage-specific exo-miRNAs. A:** ROC of all SS exo-miRNAs. **B:** expression of all SS exo-miRNAs in different stage.


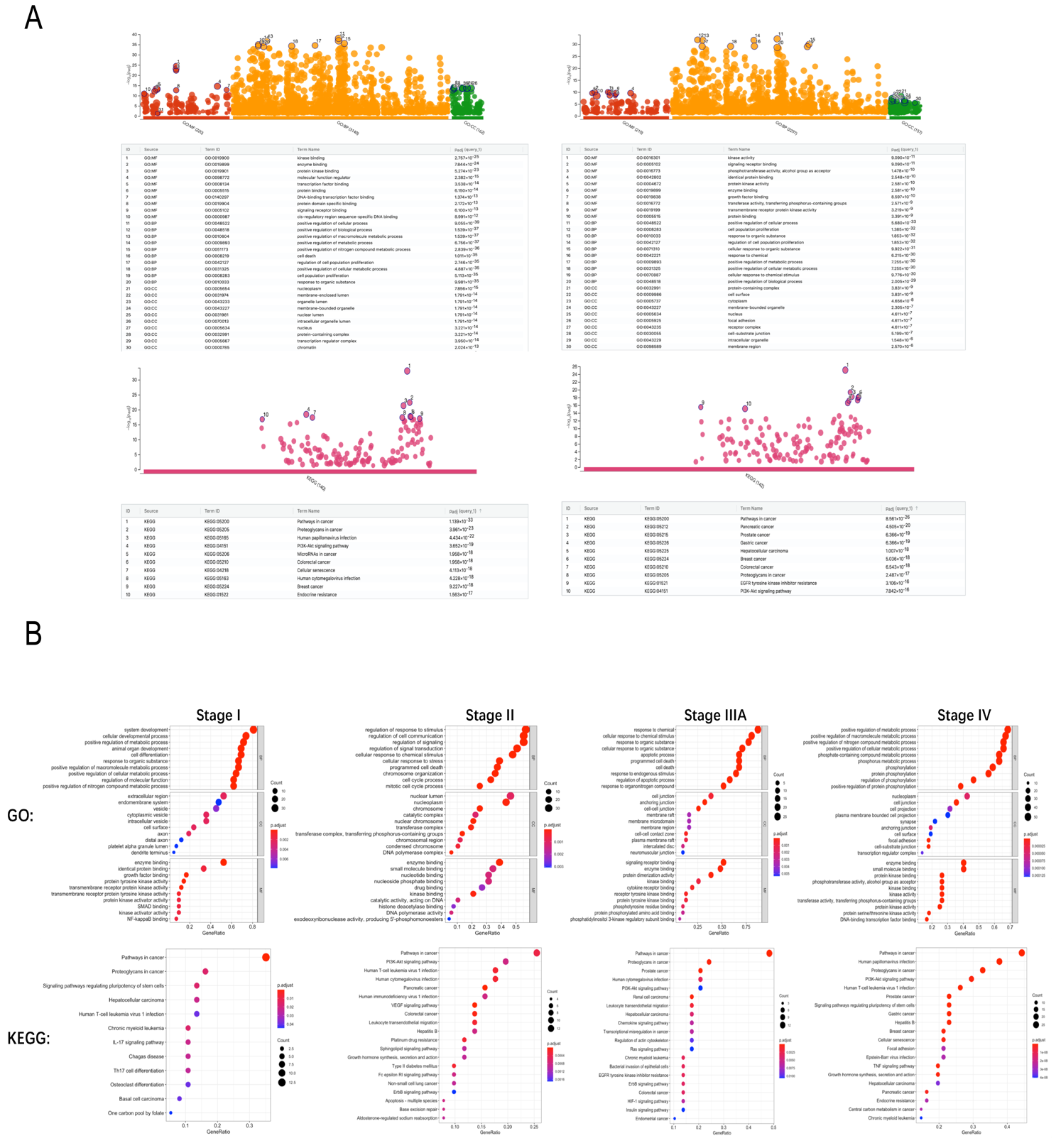


**Supplementary Figure 4|** **Enrichment analysis of stage-common and stage-specific exo-miRNAs target genes. A:** GO and KEGG enrichment analysis results of 173 hotspot genes (left) and 186 single-line regulatory genes (right) to determine whether they are enriched in biological entries and pathways associated with exosomes and cancer. **B:** GO and KEGG enrichment analysis of each stage-specific gene in five stage-specific exosomal miRNA-gene targeting networks (stage IIIB was not enriched).


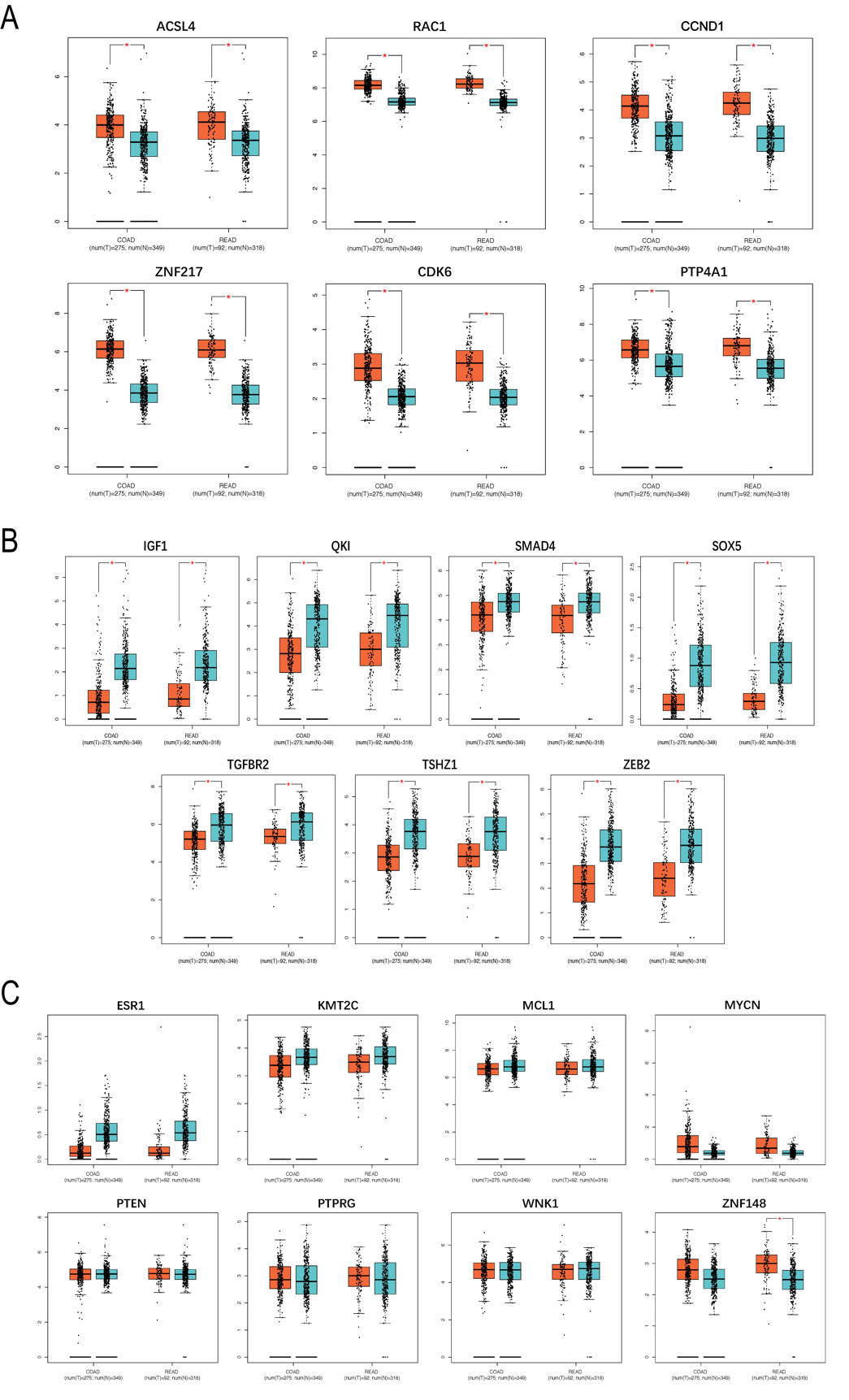


**Supplementary Figure 5|** **Expression of 26 hotspot genes in COAD and READ tissues.** Expression of 26 hotspot genes with INDEGREE > 5 in COAD and READ tissues (*, p < 0.05; **, p < 0.01; ***, p < 0.001; |logFC| > 0.5), including 10 upregulated genes (**A**, 4 others shown in the main text); 7 downregulated genes (**B**) and 9 genes at the threshold below the threshold (**C**, another 1 is shown in the main text).


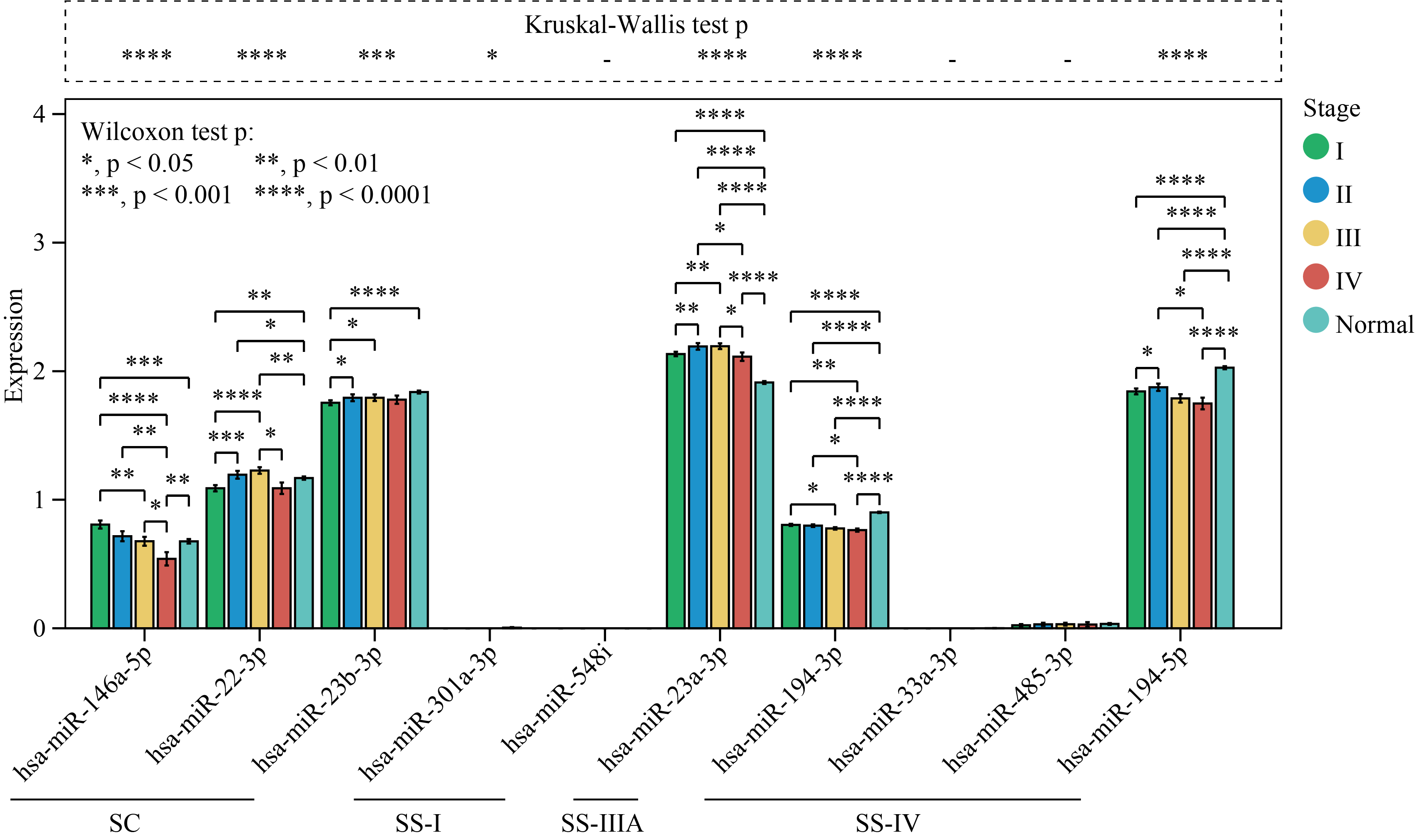


**Supplementary Figure 6| Tissue expression validation of three key SC exo-miRNAs and seven SS exo-miRNAs in GSE115513.**


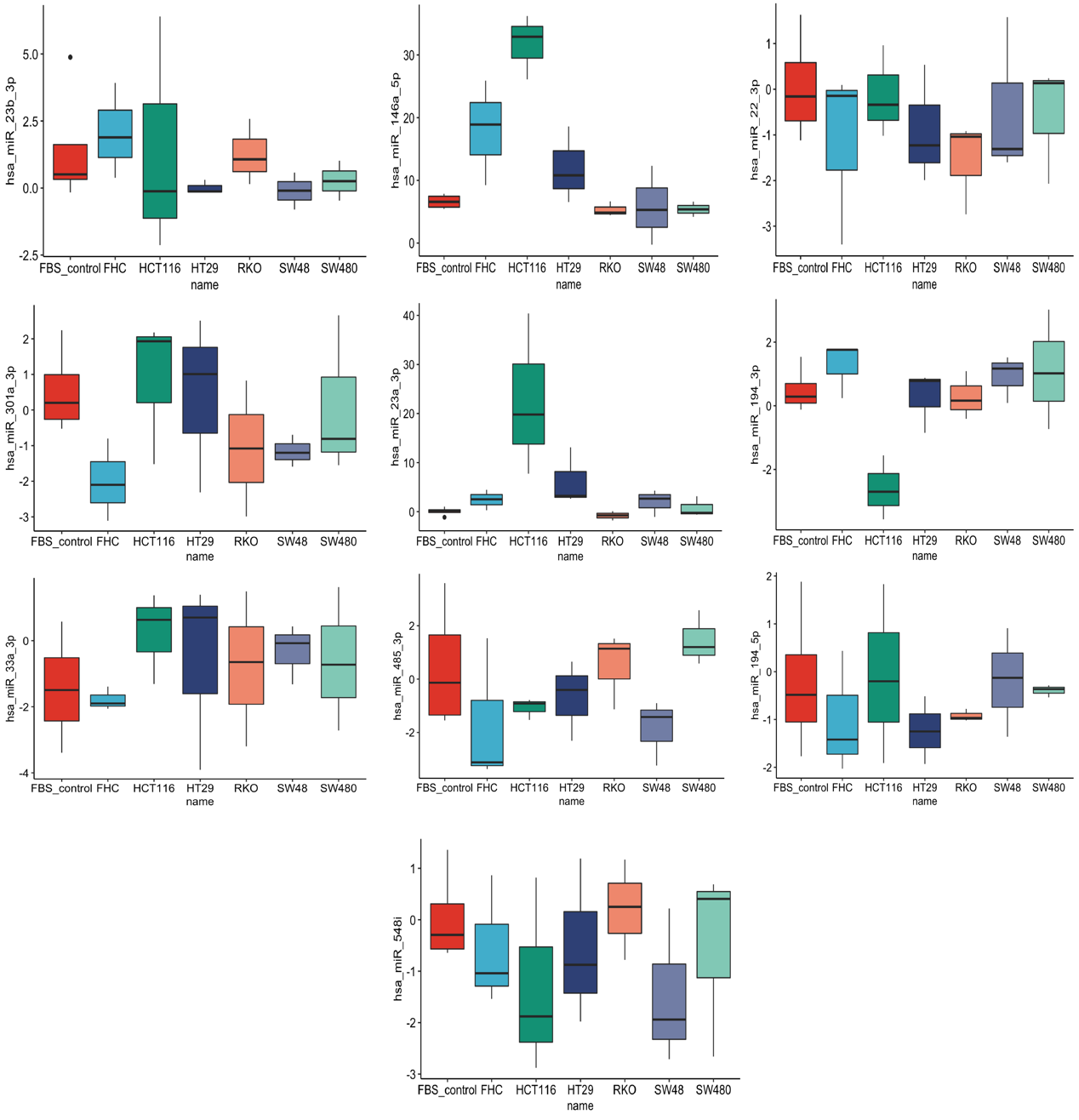


**Supplementary Figure 7|** **Expression of 10 key exo-miRNAs in exosomes of five CRC cell lines.** Expression of three CRC key exosome miRNAs and seven stage-specific exosome miRNAs in five CRC cells (HCT116, HT29, RKO, SW48, SW480) and normal colon epithelial cells (FHC) exosomes (FBS is fetal bovine serum treated control group).
